# SLUG‐related partial epithelial‐to‐mesenchymal transition is a transcriptomic prognosticator of head and neck cancer survival

**DOI:** 10.1002/1878-0261.13075

**Published:** 2021-08-21

**Authors:** Henrik Schinke, Min Pan, Merve Akyol, Jiefu Zhou, Enxian Shi, Gisela Kranz, Darko Libl, Tanja Quadt, Florian Simon, Martin Canis, Philipp Baumeister, Olivier Gires

**Affiliations:** ^1^ Department of Otorhinolaryngology, Head and Neck Surgery University Hospital LMU Munich Germany; ^2^ Department of Otorhinolaryngology The First Affiliated Hospital of Chongqing Medical University China; ^3^ School of Medicine Koç University Istanbul Turkey; ^4^ Clinical Cooperation Group ‘Personalized Radiotherapy in Head and Neck Cancer’ Helmholtz Zentrum München Neuherberg Germany

**Keywords:** head and neck squamous cell carcinoma, partial epithelial‐to‐mesenchymal transition, pEMT‐Singscore, SLUG, SNAI2

## Abstract

Partial epithelial‐to‐mesenchymal transition (pEMT) contributes to cellular heterogeneity that is associated with nodal metastases and unfavorable clinical parameters in head and neck squamous cell carcinomas (HNSCCs). We developed a single‐cell RNA sequencing signature‐based pEMT quantification through cell type‐dependent deconvolution of bulk RNA sequencing and microarray data combined with single‐sample scoring of molecular phenotypes (Singscoring). Clinical pEMT‐Singscores served as molecular classifiers in multivariable Cox proportional hazard models and high scores prognosticated poor overall survival and reduced response to irradiation as independent parameters in large HNSCC cohorts [The Cancer Genome Atlas (TCGA), MD Anderson Cancer Centre (MDACC), Fred Hutchinson Cancer Research Center (FHCRC)]. Differentially expressed genes confirmed enhanced cell motility and reduced oxidative phosphorylation and epithelial differentiation in pEMT^high^ patients. In patients and cell lines, the EMT transcription factor SLUG correlated most strongly with pEMT‐Singscores and promoted pEMT, enhanced invasion, and resistance to irradiation *in vitro*. SLUG protein levels in HNSCC predicted disease‐free survival, and its peripheral expression at the interphase to the tumor microenvironment was significantly increased in relapsing patients. Hence, pEMT‐Singscores represent a novel risk predictor for HNSCC stratification regarding clinical outcome and therapy response that is partly controlled by SLUG.

## Graphical abstract

In a combinatorial approach, a single‐cell RNA sequencing‐derived partial epithelial‐to‐mesenchymal transition (pEMT) gene signature was transferred to bulk sequencing data from large head and neck squamous cell carcinoma cohorts using single‐sample scoring (Singscoring). Patient‐specific pEMT‐SingScores prognosticated patient overall survival and reduced response to irradiation as an independent parameter. pEMT‐SingScores correlated with the expression of the transcription factor Slug, which induced characteristics of pEMT and correlated with reduced disease‐free survival.

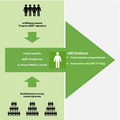

**Abbreviations**
CAFs: cancer‐associated fibroblasts
CI: confidence interval
Ctr.: control
DFS: disease‐free survival
EMT‐TF: EMT transcription factor
ESCC: esophageal squamous cell carcinoma
GSEA: gene set enrichment analysis
HNSCC: head and neck squamous cell carcinoma
HPV: human papillomavirus
HR: hazard ratio
IHC: immunohistochemistry
ITGA5: integrin alpha 5
LAMC2: laminin subunit gamma 2
LMU: Ludwig‐Maximilians University
MDACC: MD Anderson cancer cohort
OS: overall survival
OSCC: oral squamous cell carcinoma
PDPN: podoplanin
pEMT: partial epithelial‐to‐mesenchymal transition
qPCR: quantitative polymerase chain reaction
scRNAseq: single‐cell RNA sequencing
SLUG‐OE: SLUG overexpression
TCGA: The Cancer Genome Atlas program
TGFB1: transforming growth factor beta 1
ZEB1/2: zinc finger E‐box‐binding homeobox 1 and 2

## 1 Introduction

Over 600 000 patients are diagnosed yearly with head and neck squamous cell carcinoma (HNSCC) [1], which are frequently characterized by an aggressive biology and diagnosed in advanced stages. Equally aggressive, multimodal treatment is necessary that is accompanied by considerable acute and late toxicities and impaired quality of life. HNSCC is an outstandingly heterogeneous disease at the inter‐ and intratumor level [2, 3, 4, 5, 6, 7]. A major cellular differentiation program responsible for heterogeneity is the epithelial‐to‐mesenchymal transition (EMT) [8, 9, 10, 11, 12]. Mesenchymal features support tumor cells to complete various steps of the metastatic cascade and tumor progression, including local invasion, intravasation into blood and lymphatic vessels, relocalization within the patient's body via circulation, extravasation into parenchyma in locoregional and distant tissue, and survival as micrometastatic deposits [10, 13]. Accordingly, growing evidence shows that EMT traits are implicated in metastasis formation and therapy resistance *in vivo*, resulting in poor prognosis for patients [8, 10, 14]. Owing to the frequently incomplete, transitional, and reversible nature of EMT in cancer, the term partial EMT (pEMT) was coined to describe EMT in malignant cells more accurately [15, 16].

Single‐cell RNA sequencing (ScRNAseq) of a small cohort of oral cavity SCC (OSCC) confirmed a high degree of inter‐ and intratumor heterogeneity and identified a gene signature defining tumor cells in a state of pEMT [5]. Among a gene set of one hundred pEMT genes, a common pEMT signature of 15 genes was identified in carcinomas of the basal‐like subtype, which correlated with the presence of nodal metastases, poor differentiation, and other adverse pathological parameters including extracapsular extension and lymphovascular invasion in HNSCC [5, 17]. Immunohistochemical assessment of one epithelial marker (SPRRB1) and three pEMT markers (LAMC2, LAMB3, and PDPN) in tissue microarrays of OSCC patients confirmed their association with worse differentiation, perineural invasion, and nodal involvement but failed to prognosticate clinical endpoints overall (OS) and disease‐free survival (DFS) [17]. Thus, an implementation of the pEMT gene signature as a molecular classifier to predict the clinical behavior of HNSCC is lacking. Molecularly, the EMT program can be initiated by EMT transcription factors (EMT‐TFs) SNAI1 (SNAIL), SNAI2 (SLUG), TWIST, ZEB1, and ZEB2 to regulate genes related to cell adhesion, migration, and invasion [18, 19, 20]. Specific roles of these EMT‐TFs in the regulation of pEMT in HNSCC remain poorly explored.

There is a strong medical need for a biomarker‐based selection of treatment modalities in HNSCC. Reliable assessment of the risk of treatment resistance and locoregional spread holds potential to spare unnecessary radical resections of primary carcinomas, unneeded lymph node dissections, and/or systemically administered radio(chemo)therapy for the selected patients. Oppositely, we need to recognize patients who should be treated more aggressively to avoid recurrence and metastasis at earlier stages of disease. Moreover, a profound understanding of the mechanisms involved in therapy resistance and metastasis formation provides opportunities to identify new therapeutic options. Therefore, the present study investigated the relevance and regulators of pEMT as a prognosticator in clinical HNSCC cohorts.

## 2 Methods

### 2.1 Data analysis

Data analysis was performed using r ([21]; R version 3.6.1 (2019‐07‐05)). Correlation matrices were calculated with cran *corrplot*. Univariable and multivariable survival analyses were performed with cran *survival* and *survminer*. Univariables were subjected to Cox proportional hazard (CoxPH) models, and significant univariables according to Wald test *P*‐value were implemented in multivariable CoxPH models. Further analysis was performed using cran *tidyverse*.

### 2.2 Bulk RNA‐Seq and EPIC deconvolution

For The Cancer Genome Atlas (TCGA), expression profiles of 18 464 protein‐coding genes from the whole human genome were extracted. r‐package *CGDS* (bioconductor) was used to extract expression profiles and clinical data (cancer study: ‘hnsc_tcga_pan_can_atlas_2018’/case list: ‘hnsc_tcga_pan_can_atlas_2018_all’/genetic profile: ‘hnsc_tcga_pan_can_atlas_2018_rna_seq_v2_mrna’) for *n* = 415 HPV‐negative TCGA HNSCC patients.

MD Anderson Cancer Centre (MDACC) data were retrieved from GEO object GSE42743 with r bioconductor packages *GEOquery, affy, AnnotationDbi*, and *hgu133plus2.db* to extract and map microarray expression data and receive corresponding clinical data. *affy* function *rma* for robust multi‐array average expression measure with default settings was applied to MDACC data to receive log2‐transformed data. From the 18 464 protein‐coding genes found in TCGA, *n* = 16 965 were recovered in the MDACC data set for *n* = 73 MDACC HNSCC patients.

Fred Hutchinson Cancer Research Center (FHCRC) data were retrieved from GEO object GSE41613 without further processing. From the 18 464 protein‐coding genes found in TCGA, *n* = 15 794 were recovered in the FHCRC data set for *n* = 97 FHCRC HNSCC patients.

The ‘Estimating the Proportion of Immune and Cancer cells’ (EPIC) algorithm was used within RNAseq and microarray expression bulk data from TCGA, MDACC, and FHCRC. For MDACC and FHCRC, normalized mRNA expression data were squared prior to EPIC deconvolution. Samples with a robust convergence value (‘*fit.gof$convergenceCode’* = 0) were obtained for *n* = 375 (TCGA), *n* = 62 (MDACC), and *n* = 85 patients (FHCRC).

### 2.3 pEMT‐Singscores

For TCGA, expression data were log2‐transformed prior to calculations. Using the r‐package *singscore* [22] (bioconductor), a Singscore was computed for each TCGA, MDACC, and FHCRC patient with the common *n* = 15 pEMT genes defined by Puram *et al*. [5]. Respective numbers of protein‐coding genes (see previous paragraph) in gene ranks served as a genetic background.

### 2.4 Patient selection after EPIC deconvolution

To determine the impact of the different cellular proportions estimated by EPIC on p‐EMT‐Singscores, a stepwise model selection was used. All results from EPIC (‘Bcells’, ‘CAFs’, ‘CD4_Tcells’, ‘CD8_Tcells’, ‘Endothelial’, ‘Macrophages’, ‘NKcells’, ‘otherCells’) and the pEMT‐Singscore, as the response value, were applied to *stepwise* from cran package *RcmdrMisc*. To stepwise built the best performing linear model, ‘backward/forward’ was chosen as direction and AIC as selection criterion. Cell types selected by *stepwise* were applied to hierarchical clustering using ward distance. Clustering with heatmaps revealed a distortion of pEMT‐Singscores by CAFs, which was further confirmed by Spearman correlation analysis. In TCGA, a cluster with CAF proportions of 48.3% or more was visualized and excluded from further analysis. Eventually, *n* = 303 (TCGA), *n* = 62 (MDACC), and *n* = 85 (FHCRC) patients were selected.

### 2.5 Survival analysis

For univariable survival analysis, CoxPH ratios (HR) > 1 with Wald *P*‐value ≤ 0.05 were accepted as relevant. For visualization, Singscores were implemented to dichotomize patients into 40% lowest (‘low’) and 40% highest groups (‘high’) in analogy to Puram *et al*. [5]. Then, a CoxPH model, median survival times, and logrank *P*‐values were calculated and included in Kaplan–Meier curves.

### 2.6 Differential gene expression

Differentially expressed genes (DEGs) between pEMT‐low and pEMT‐high patients in TCGA, MDACC, and FHCRC were analyzed with bioconductor packages *DESeq2, edgeR*, and *limma*.*voom* (or *limma* for microarray samples of MDACC and FHCRC). pEMT‐low patients served as baseline and pEMT‐high patients as comparison. For TCGA, raw expression values without log2 transformation were applied to DEG analysis. Squared expression data from MDACC and FHCRC were applied to DEG analysis.

### 2.7 Gene set enrichment analysis

Gene set enrichment analysis (GSEA) in GO terms Biological Process was conducted using resulting DEGs from *edgeR*. *edgeR* functions *goana* and *topGO* were conducted to determine top 20 up‐ and down‐regulated GO terms in all cohorts with a false discovery rate cutoff of 0.05. *goana* uses the NCBI RefSeq annotation, and Entrez gene identifiers were mapped to gene symbols with *org.Hs.eg*.*db*.

### 2.8 Cancer Cell Line Encyclopedia

Data from the Cancer Cell Line Encyclopedia (CCLE) were downloaded from the Broad Institute (‘https://data.broadinstitute.org/ccle/’). ‘CCLE_RNAseq_rsem_genes_tpm_20180929.txt’ was processed in r. Cell line data from ‘UPPER_AERODIGESTIVE_TRACT’ and ‘OESOPHAGUS’ were extracted. Expression data from *n* = 17 759 human protein‐coding genes could be extracted for *n* = 58 cell lines and were used to compute pEMT‐Singscores.

### 2.9 Ethics approval and consent to participate

Clinical samples of the LMU HNSCC cohort were obtained after written informed consent during routine surgery, based on the approval by the ethics committee of the local medical faculties (Ethikkommission der Medizinischen Fakultät der LMU; 087–03; 197–11; 426–11, EA 448‐13, and 17‐116) and in compliance with the WMA Declaration of Helsinki and the Department of Health and Human Services Belmont Report.

### 2.10 Human samples

The Ludwig‐Maximilians‐Universität (LMU) HNSCC cohort included HPV‐negative tumor specimens from *n* = 76 patients [23]. Primary tumor extent was evaluated during diagnostic pan‐endoscopies, and synchronous second primary tumors of the upper aerodigestive tract were excluded. Biopsies were taken to confirm suspected diagnoses and pretherapeutic imaging completed the staging. Surgical resection of carcinomas with or without neck dissection and, if necessary, reconstructive measures were recommended as primary treatment by a multidisciplinary tumor board. Depending on postoperative histopathologic findings, adjuvant therapy was advised by the tumor board according to the guidelines of the National Comprehensive Cancer Network (NCCN; https://www.nccn.org/guidelines). In addition to the resection of the primary carcinoma, four patients underwent ipsilateral neck dissection, 60 had bilateral neck dissection, and 12 patients had no additional surgery of the neck. Twenty‐four patients had adjuvant radiotherapy; 26 were treated by chemoradiation and two by immuneradiation. Radiation dose varied between 54 and 70 Gray (Gy) with an average of 64.5 Gy and a median of 64.0 Gy. In the remaining 24 cases, either no adjuvant treatment was recommended, or patients rejected recommendations. Samples of the primary carcinoma were cryopreserved by snap‐freezing in tissue‐Tek^®^ (Sakura, Finetek, the Netherlands) and processed to 5‐µm‐thick sections for further staining as has been described [24].

### 2.11 Immunohistochemistry scoring and immunofluorescence

Antibodies specific for SLUG (C19G7; Cell Signaling Technology, NEB, Frankfurt, Germany, #9585, 1 : 400), pan‐cytokeratine (polyclonal; Invitrogen, Camarillo, CA, USA, #18‐0059, 1 : 200), EGFR (Dianova, Hamburg, Germany, #DLN‐08892, 1 : 200), LAMC2 (Novus Biologicals, Centennial, CO, USA, 1 : 1000), and E‐cadherin (24E10, Cell Signaling Technology, NEB, #3195, 1 : 400) were used for immunohistochemistry (IHC) and immunofluorescence (IF) staining the with avidin–biotin–peroxidase method (Vectastain, Vector Laboratories, Burlingame, CA, USA) or Alexa Fluor‐488‐conjugated secondary antibody. Confocal microscopy images were recorded with a TCS‐SP5 system (Leica Microsystems, Wetzlar, Germany). IHC scores were assessed by two experienced scorers independently in blinded manner as described [25].

### 2.12 Cell lines and treatments

FaDu, Kyse30, Cal27, and Cal33 cell lines were obtained from ATCC (Manassas, VA, USA) and DSMZ and were confirmed by STR typing using 10 makers (AMEL, CSF1PO, D13S317, D16S539, D21S11, D5S818, D7S820, TH01, TPOX, vWA; Table S1). Kyse30 and FaDu cells were stably selected with SLUG‐Myc in the 141‐pCAG‐3SIP vector with MATra (PromoCell, Heidelberg, Germany) using 1 µg·mL^−1^ puromycin (Sigma, Taufkirchen, Germany). Control cell lines were transfected with 141‐pCAG‐3SIP. Cells were maintained in RPMI 1640 or DMEM, 10% FCS, 1% penicillin/streptomycin, in a 5% CO_2_ atmosphere at 37 °C.

### 2.13 Western blotting

Cell lysates were extracted as described [23]. Ten to fifty micrograms of proteins was separated by 10% SDS/PAGE and visualized with primary SLUG or E‐cadherin antibody (C19G7; Cell Signaling Technology, #9585, 1 : 1000; 24E10, Cell Signaling Technology, #3195, 1 : 1000) and horseradish peroxidase (HRP)‐conjugated secondary antibodies (1 : 5000), and ECL reagent (Millipore, Darmstadt, Germany) in a Chemidoc XRS imaging system (Bio‐Rad, Munich, Germany). An HRP‐conjugated antibody was used to visualize beta‐actin (sc‐47778 HRP; Santa Cruz Biotechnology, Santa Cruz, CA, USA).

### 2.14 Reverse transcription qPCR analysis

RT‐qPCR was performed and quantified as described [23]. Quantifications exceeding a cycle threshold (CT) of 35 were regarded as not expressed.

E‐CADHERIN‐FW 5′‐TGC CCA GAA AAT GAA AAA GG‐3′
E‐CADHERIN‐BW 5′‐GTG TAT GTG GCA ATG CGT TC‐3′
GAPDH‐FW 5′‐AGG TCG GAG TCA ACG GAT TT‐3′
GAPDH‐BW 5′‐TAG TTG AGG TCA ATG AAG GG‐3′
ITGA5‐FW 5′‐GGCTTCAACTTAGACGCGGAG‐3′
ITGA5‐BW 5′‐TGGCTGGTATTAGCCTTGGGT‐3′
LAMC2‐FW 5′‐CAAAGGTTCTCTTAGTGCTCGAT‐3′
LAMC2‐BW 5′‐CACTTGGAGTCTAGCAGTCTCT‐3′
MMP10‐FW 5′‐TCAGTCTCTCTACGGACCTCC‐3′
MMP10‐BW 5′‐CAGTGGGATCTTCGCCAAAAATA‐3′
PDPN‐FW 5′‐ACCAGTCACTCCACGGAGAAA‐3′
PDPN‐BW 5′‐GGTCACTGTTGACAAACCATCT‐3′
TGFB1‐FW 5′‐CTTCGCCCCTAGCAACGAG‐3′
TGFB1‐BW 5′‐TGAGGGTCATGCCGTGTTTC‐3′
SLUG‐FW 5′‐TGA TGA AGA GGA AAG ACT ACAG‐3′
SLUG‐BW 5′‐GCT CAC ATA TTC CTT GTC ACA G‐3′
SNAIL‐FW 5′‐GCG AGC TGC AGG ACT CTA AT‐3′
SNAIL‐BW 5′‐CCT CAT CTG ACA GGG AGG TC‐3′
VIMENTIN‐FW 5′‐GAG AAC TTT GCC GTT GAA GC‐3′
VIMENTIN‐BW 5′‐GCT TCC TGT AGG TGG CAA TC‐3′
ZEB1‐FW 5′‐TGC ACT GAG TGT GGA AAA GC‐3′
ZEB1‐BW 5′‐TGG TGA TGC TGA AAG AGA CG‐3′

### 2.15 Annexin V‐FITC and propidium iodide staining

Annexin V‐FITC and propidium iodide (PI) dual staining kit (Invitrogen, eBioscience Annexin V‐FITC Apop, eBioscience Thermo Fisher Scientific, Munich, Germany, BMS500FI‐300) were used according to the manufacturer's protocol. Cells were analyzed by flow cytometry (Beckman‐Coulter, CytoFLEX, Krefeld, Germany). Flow cytometry gates were chosen based on untreated controls for each cell line, and values were normalized to controls.

### 2.16 Cell proliferation assay

Cells were counted using a Leica DMi8 microscope (Leica, Wetzlar, Germany) with las x software (Leica Microsystems, Wetzlar, Germany) and fiji [26]. In a 96‐well, 2000 cells were seeded and left overnight to fully adhere. The next day (timepoint 0 h), and 24 or 48 h later (timepoints 24 and 48 h) were measured. Cells were stained 15 min with Hoechst 33342 (ThermoFisher, Munich, Germany). Using las x software, 72 images per well with 100× magnification were taken and merged. In fiji, images in grayscale (16‐bit) were compressed to 8 bit by threshold adjustment to remove noise. Watershed function was applied to cut any artificially merged pixels. Resulting particles, representing single cells, were counted and summarized. Resulting counted cell numbers were then analyzed using r [21].

### 2.17 Fibroblast spheroid invasion assay and Matrigel invasion assay

Spheroids of normal human foreskin fibroblasts (PromoCell, C‐12352, Heidelberg, Germany) were grown in ultra‐low attachment plates over 24 h by seeding 1 × 10^4^ cells in standard DMEM. Following fibroblast spheroid formation, 1 × 10^4^ FaDu or Kyse cells were added and co‐cultured for additional 48 and 72 h. Co‐cultured spheroids were carefully harvested and frozen in tissue‐Tek (Sakura Europe) in a cryomold with liquid nitrogen. Cryosections of 5 µm thickness were generated for IHC. Matrigel invasion assays were conducted in accordance with Shaw [27].

### 2.18 Clonogenic survival assay

1 × 10^3^ FaDu and 5 × 10^3^ Kyse30 cells were plated on a 6‐well plate and irradiated the next day. Irradiation was performed using a CIX2 cabinet irradiator (Xstrahl, Camberley, UK) equipped with a 0.5‐mm Cu filter. Irradiation was conducted as a single irradiation dose of 2 or 10 Gy, or as a fractionated irradiation of five times 2 Gy ever 24 h. After 14 days for FaDu and 10 days for Kyse30, cells were fixed and stained with crystal violet solution containing methanol. Six‐well plates were photographed using Chemidoc XRS. colonyarea imagej Plugin by Guzmán *et al*. [28] was used to quantify colony areas. Clonogenic survival was calculated by measuring the area of colonies of irradiated relative to nonirradiated control plates.

## 3 Results

### 3.1 Cohort deconvolution and pEMT quantification

**Fig. 1:**
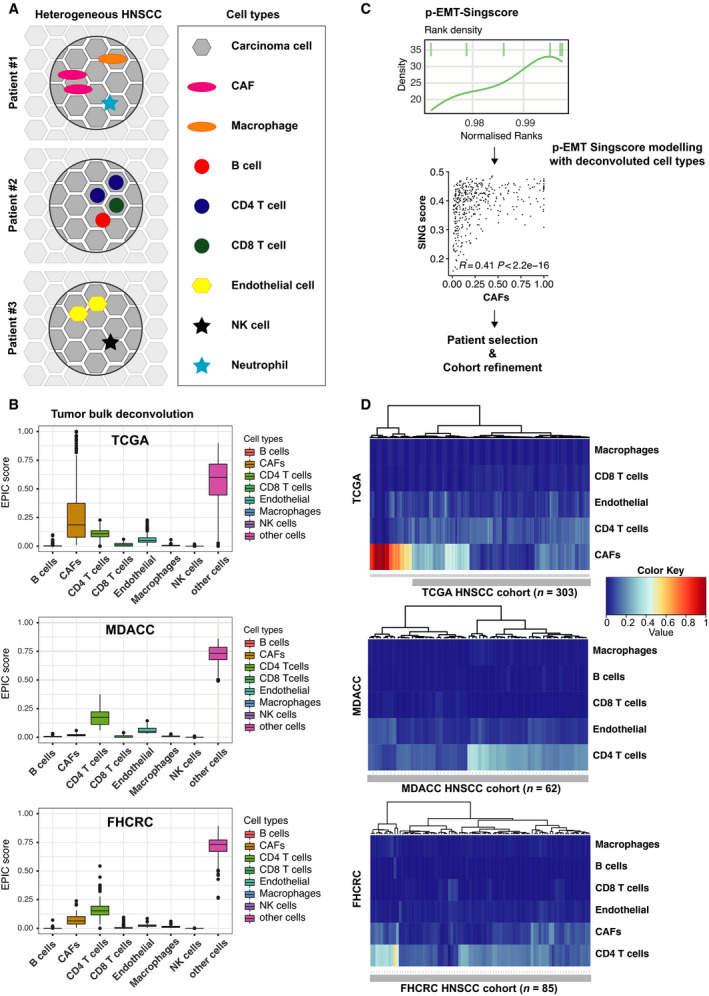
Generation of pEMT‐Singscores. (A) Schematic representation of cellular heterogeneity in HNSCC including nonmalignant cell types. (B) Bulk RNA‐seq deconvolution using the EPIC algorithm. Shown are proportions of the indicated cell types in TCGA, MDACC, and FHCRC. (C) Schematic representation of pEMT‐Singscore generation, stepwise feature selection, and determination of nonmalignant cell types modeling pEMT for patient selection and cohort refinement. (D) Heatmap with hierarchical clustering of Euclidean distance matrix of patients of the TCGA, MDACC, and FHCRC cohorts for the indicated cell types implemented in the modeling of pEMT‐Singscores. Refined cohorts lacking patients with high CAF content in TCGA are marked with a gray bar and patient numbers are indicated.

Tumor biopsies in large clinical cohorts may vary in their composition of malignant and nonmalignant cells (schematic representation in Fig. 1A). The latter ones can contribute unwanted gene expression profiles of mesenchymal cells and thus bias the measurement of pEMT in bulk RNAseq and microarray datasets [5]. HPV‐negative HNSCC patients of TCGA (*n* = 415), MD Anderson Cancer Center (MDACC; *n* = 73), and Fred Hutchinson Cancer Research Center (FHCRC; *n* = 97) cohorts were subjected to cell type deconvolution using the EPIC algorithm [29]. Reliable proportions of cancer‐associated fibroblasts (CAFs), macrophages, B cells, CD4/CD8 T cells, endothelial cells, natural killer cells (NK cells), neutrophils, and tumor cells were computed for *n* = 375, *n* = 62, and *n* = 85 patients in TCGA, MDACC, and FHCRC, respectively. Cohorts contained samples with > 65% of carcinoma cells and > 10% of CD4 T cells, and TCGA was further characterized by substantial proportions of CAFs, including single samples with CAF contents exceeding 75% (Fig. 1B).

pEMT‐Singscores were generated based on the relative expression of 15 common pEMT genes against the background of 18 464, 16 965, and 15 794 protein‐coding genes expressed in TCGA, MDACC, and FHCRC. pEMT‐Singscores represent patient‐specific, single‐sample gene set enrichments defining the degree of pEMT of individual patients. pEMT‐Singscores were modeled using stepwise feature selection including estimated proportions of all *n* = 9 nonmalignant cell types. Cell types selected by stepwise modeling and best describing pEMT‐Singscores were used to generate hierarchical clusters of patients and exclude patients with high proportions of pEMT‐contributing nonmalignant cells. Spearman correlations of single‐cell types disclosed high positive correlations of pEMT‐Singscores with CAFs in all cohorts (Fig. 1C and Fig. S1). TCGA patients in the cluster with high proportions of CAFs were therefore excluded from further analysis resulting in *n* = 303, *n* = 62, and *n* = 85 selected patients from TCGA, MDACC, and FHCRC, respectively (Fig. 1D).

### 3.2 pEMT‐Singscore is an independent prognostic factor in HNSCC

pEMT‐Singscores were re‐assessed following patient selection and followed normal distributions in all cohorts (Fig. S2A–C, upper left panels). Plotting of normalized ranks of *n* = 15 common pEMT genes displayed a more widespread density for the specific patient with the single lowest pEMT‐SingScore, whereas it showed a stronger accumulation of the genes toward higher ranks in the specific patient with the single highest pEMT‐SingScore (Fig. S2A–C, lower panels). This accumulation was further substantiated in a dispersion plot, where high pEMT‐Singscores correlated with low dispersion of normalized ranks (Fig. S2A–C, upper right panels).

**Fig. 2:**
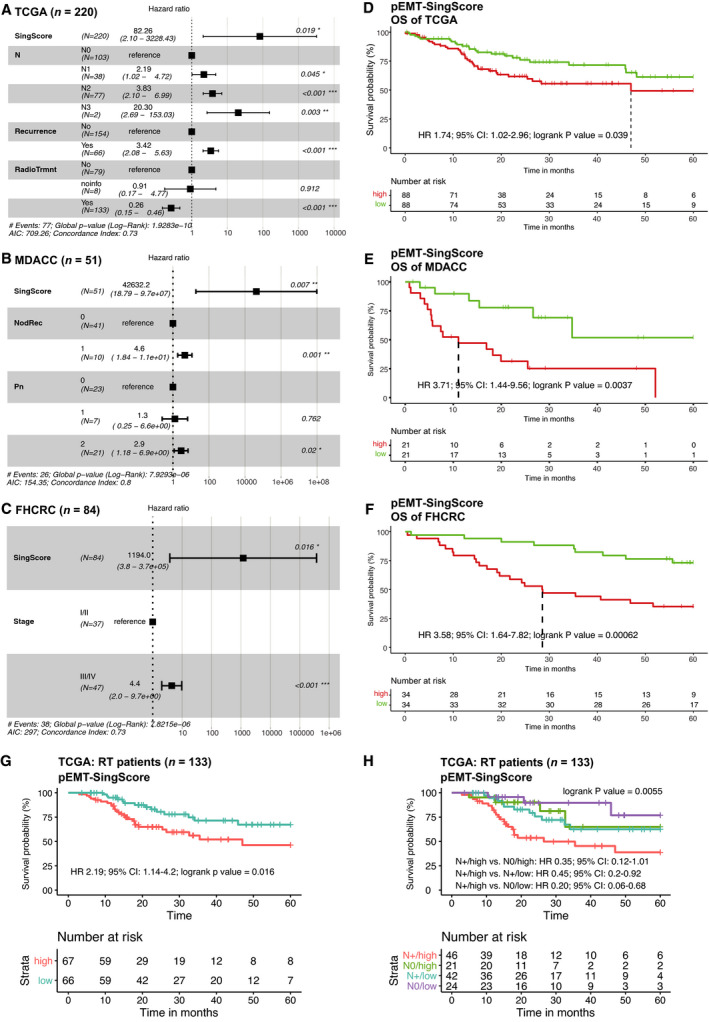
pEMT‐Singscore is a molecular prognosticator of OS in HNSCC. (A–C) pEMT‐Singscores were calculated for *n* = 15 common pEMT genes for patients in the TCGA (A), MDACC (B), and FHCRC (C) cohorts and served to compute a multivariable CoxPH model. Shown are Forest plots including all univariables significantly associated with OS in multivariable CoxPH models with patient numbers, linear hazard ratios, 95% CI, logrank *P*‐values, AIC, and concordance indexes. (D–F) Kaplan–Meier curves stratified after pEMT‐Singscores with CoxPH ratio (HR), 95% confidence intervals (CI), logrank *P*‐values, and survival table is shown. pEMT‐Singscore^high^: upper 40%; pEMT‐Singscore^low^: lower 40%. (G, H) Kaplan–Meier curve stratified after median pEMT‐Singscores with CoxPH ratio (HR), 95% confidence intervals (CI), logrank *P*‐values, and survival table is shown for *n* = 133 patients of the TCGA cohort who received therapeutic irradiation. In (H), patients are further stratified according to the presence and absence of nodal metastases (N+/N0).

Variables significantly associated with OS in univariable CoxPH models (see Tables [Link], [Link], [Link] for univariables, HR, 95% CI, and *P*‐values) were implemented in a multivariable CoxPH model. pEMT‐Singscores were significantly associated with OS in univariable analysis and were identified as independent prognostic marker of OS in multivariable CoxPH models for all three HNSCC cohorts (Fig. 2A–C, TCGA: HR: 82.26, 95% CI: 2.10–3228.43, logrank *P* = 0.0019; MDACC: HR: 42 632.2, 95% CI: 18.79–9.7e+7, logrank *P* = 0.007, FHCRC: HR: 1194.0, 95% CI: 3.8–3.7e+5, logrank *P* = 0.016). Additional independent prognosticators were nodal status, recurrence, and irradiation (TCGA; *n* = 220), nodal and local recurrence, extracapsular spread, and perineural invasion (MDACC; *n* = 51), and tumor stage (FHCRC; *n* = 84) (Fig. 2A–C).

Overall survival was significantly decreased in pEMT‐Singscore^high^ patients (upper 40%) compared to pEMT‐Singscore^low^ patients (lower 40%) in all three cohorts (Fig. 2D–F, TCGA: HR: 1.74, CI: 1.02–2.96, logrank *P* = 0.039; MDACC: HR: 3.71, CI: 1.44–9.56, logrank *P* = 0.00037; FHCRC: HR: 3.58, CI: 1.64–7.82, logrank *P* = 0.00062). An alpha error of 6.8% for pEMT‐Singscores was assessed through 10 000 calculations with 15 different randomly selected genes from the TCGA gene pool (18 464 genes excluding all 15 common pEMT genes [5]), demonstrating a high specificity of the pEMT‐Singscore in the prognosis of OS.

Next, TCGA patients who received therapeutic irradiation were extracted (*n* = 133). Variables significantly associated with OS were determined in univariable CoxPH models and were implemented in multivariable CoxPH modeling. This identified pEMT‐Singscores, nodal status, and recurrences as independent prognostic factors of OS for irradiated patients in the TCGA cohort (Fig. S2D). OS following radiotherapy was significantly decreased in pEMT‐Singscore^high^ patients compared with pEMT‐Singscore^low^ patients (Fig. 2G, HR 2.19, 95% CI: 1.14–4.2, logrank *P*‐value = 0.016). Further stratification according to nodal status of irradiated patients demonstrated significant differences (logrank *P*‐value = 0.0055). Patients without nodal involvement and low pEMT‐Singscores had highest survival probabilities, whereas patients with lymph node metastases and a high degree of pEMT had the poorest survival probabilities (Fig. 2H). Irradiated patients without nodal involvement but high pEMT and patients with lymph node metastases but low pEMT did not differ in OS after five years (Fig. 2H). Hence, pEMT‐Singscores represent an independent significant prognosticator of OS and response to irradiation in HNSCC patients that have an impact comparable to nodal metastases.

### 3.3 pEMT‐associated gene regulation

**Fig. 3:**
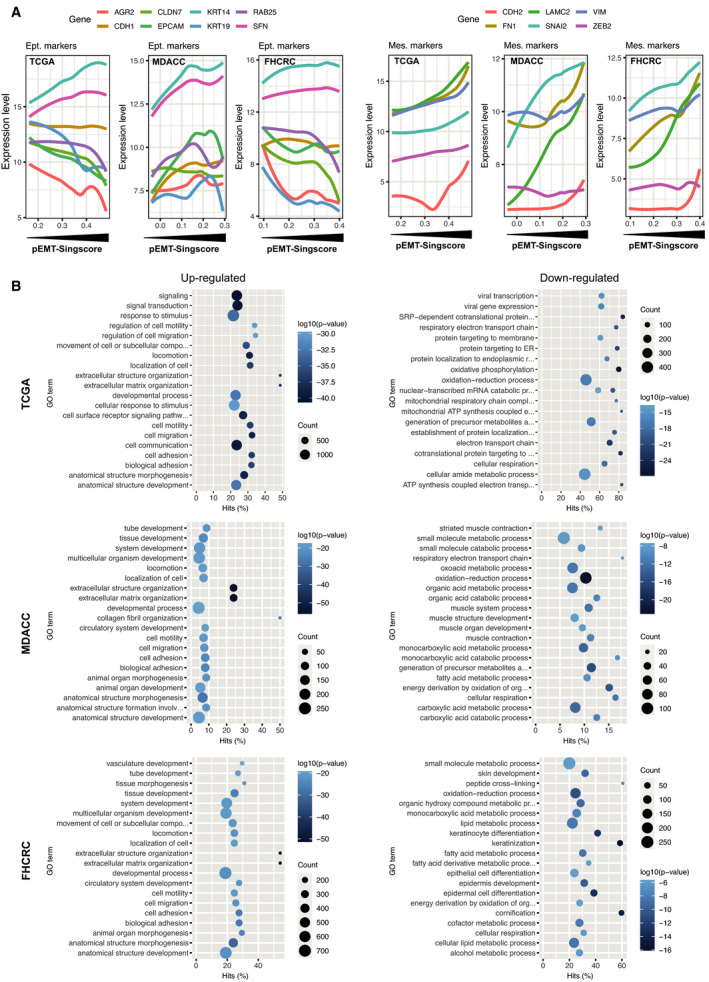
Gene expression profiles in pEMT (A) Expression levels of epithelial (E‐cadherin, claudin 7, keratin 14 and 19, Rab25, EpCAM, anterior gradient 2, stratifin 1) and mesenchymal markers (fibronectin 1, vimentin, N‐cadherin, laminin C2), and the EMT‐TFs SLUG and Zeb2 in the TCGA, MDACC, and FHCRC cohorts were plotted with line smoothing using loess against pEMT‐Singscores. (B) DEGs were identified using DESeq, edgeR, and limma‐voom algorithms comparing pEMT‐Singscore^high^ and pEMT‐Singscore^low^ patients in the TCGA, MDACC, and FHCRC cohorts. GSEA of GO terms biological process was conducted and the top *n* = 20 (log10 *P*‐value) up‐ and down‐regulated GO terms are depicted including gene counts.

To address whether pEMT‐Singscores can quantify an incomplete transition of cells to a more mesenchymal state while retaining major epithelial features, expression of selected epithelial and mesenchymal markers was correlated with pEMT‐Singscores in TCGA, MDACC, and FHCRC. In TCGA and FHCRC, epithelial markers (anterior gradient 2, claudin 7, Rab25, E‐cadherin, EpCAM, keratin 19) remained constant or were reduced with increasing pEMT‐Singscores, whereas keratin 14 and stratifin were moderately increased (Fig. 3A). In MDACC, keratin 14 and stratifin increased with pEMT‐Singscores, while the remaining epithelial markers showed fluctuating expression (Fig. 3A). Mesenchymal markers N‐cadherin, laminin C2, vimentin, fibronectin 1, and SLUG (SNAI2) were enhanced with increasing pEMT‐Singscores in all three cohorts, while ZEB2 remained constant (Fig. 3A).

pEMT‐Singscores were computed from bulk RNAseq datasets of cell lines of the upper aerodigestive tract [ESCC (esophageal squamous cell carcinoma) and HNSCC] available from the CCLE database. pEMT‐Singscores inversely correlated with E‐cadherin and EpCAM and directly correlated with fibronectin and vimentin (Fig. S3). Direct correlation of pEMT‐Singscores was also observed with EGFR and N‐cadherin but did not reach statistical significance (Fig. S3).

Differentially regulated genes (DEGs) were assessed with *DESeq2*, *edgeR*, and *limma‐voom* (RNAseq) or *limma* (microarray data) algorithms in the TCGA, MDACC, and FHCRC cohorts. pEMT‐Singscore^low^ patients (lower 40%) served as baseline to compare with pEMT‐Singscore^high^ patients (upper 40%) in analogy to Puram *et al*. [5]. DEGs identified by all three algorithms in all three cohorts represented *n* = 92 genes with *n* = 89 up‐regulated and *n* = 3 down‐regulated genes. DEGs included no cell cycle gene, 34 pEMT genes, no epithelial differentiation type 1 gene, no epithelial differentiation type 2 gene, one stress gene, and four hypoxia genes according to single‐cell signatures described by Puram *et al*. [5] (Table S5). Up‐regulated GO terms were primarily related to cell motility and migration in all three cohorts (Fig. 3B). Down‐regulated GO terms were related to oxidative phosphorylation, metabolic processes, and epithelial, epidermis, and keratinocyte differentiation (Fig. 3B). Thus, pEMT‐Singscores reflect partial changes of tumor cells toward a more mesenchymal gene expression profile that is associated with cellular locomotion at the transcriptome level.

### 3.4 SLUG correlates with pEMT and induces a pEMT phenotype, invasion, and enhanced resistance to irradiation

**Fig. 4:**
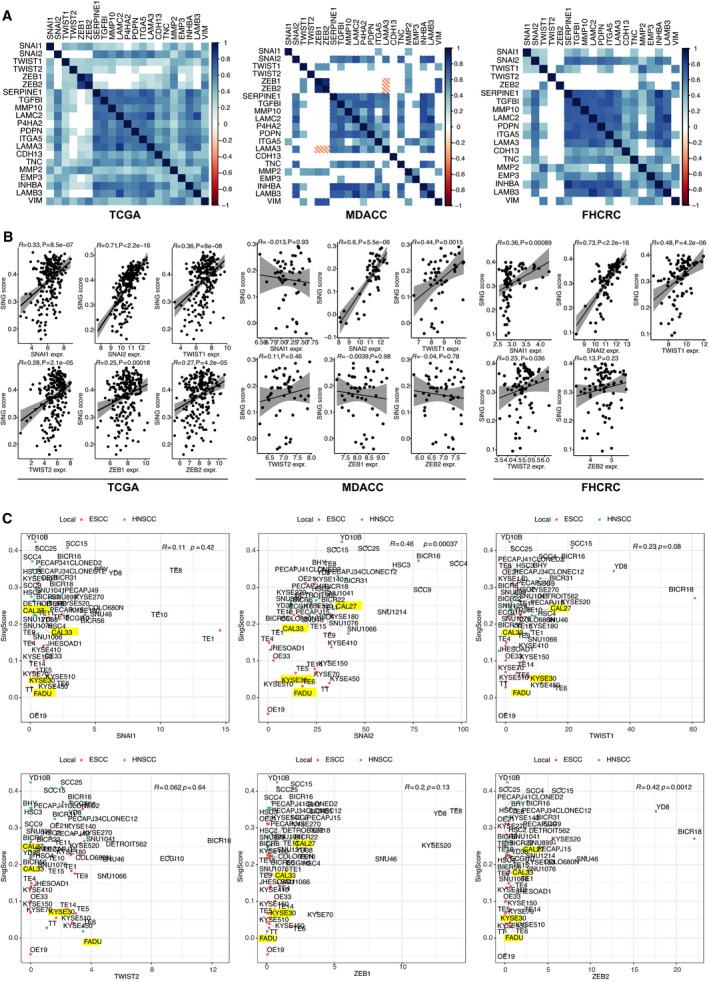
EMT‐TF SLUG positively correlates with pEMT‐Singscores. (A) Pearson correlation matrices of Snail, SLUG, Twist 1, Twist2, Zeb1, and Zeb2 with *n* = 15 common pEMT genes in the TCGA, MDACC, and FHCRC cohorts. Heatmap encodes Pearson’s *r*. Insignificant values are blank. Significance level ≤ 0.01. (B) Spearman’s rank correlation of Snail, SLUG, Twist 1, Twist2, Zeb1, and Zeb2 with pEMT‐Singscores in the TCGA, MDACC, and FHCRC cohorts. Spearman’s rho with *P*‐values is depicted. (C) pEMT‐Singscores were computed in esophageal (ESCC) and HNSCC cell lines of the CCLE database and are plotted against expression values of Snail, SLUG, Twist 1, Twist2, Zeb1, and Zeb2. ESCC and HNSCC cell lines are depicted as red and blue dots, respectively. Cell lines further investigated *in vitro* are marked as yellow. Spearman’s rho and *P*‐values are indicated.

Aiming at identifying potential regulators of pEMT in HNSCC, expression of Snail, SLUG, Twist1, Twist2, Zeb1, and Zeb2 was correlated with the 15 common pEMT genes in TCGA, MDACC, and FHCRC. Strongest positive correlation of EMT‐TFs with single genes of the common pEMT signature was observed for SLUG (Fig. 4A). In all cohorts, SLUG showed the strongest correlation with pEMT‐Singscores (TCGA: rho = 0.71; *P* < 2.2e‐16; MDACC: rho = 0.65; *P* = 4.7e‐08; FHCRC: rho = 0.73; *P* < 2.2E‐16). All other EMT‐TFs were characterized by substantially inferior correlations (Fig. 4B) and SLUG was the only EMT‐TF comprised in up‐regulated DEGs (Table S5). pEMT‐Singscores were computed in ESCC and HNSCC cell lines of CCLE using 17 760 protein‐coding genes and were correlated to EMT‐TFs. In confirmation of clinical samples, ESCC and HNSCC cell lines displayed highest correlations of pEMT‐Singscores with SLUG (rho = 0.46; *P* = 0.00037) (Fig. 4C).

A potential involvement of SLUG in pEMT regulation was analyzed *in vitro* in cell lines of the upper aerodigestive tract, that is, FaDu (HNSCC) and Kyse30 (ESCC). These cell lines induce EMT following treatment with high‐dose EGF, which was associated to the activation of SLUG transcription [23]. Unlike oral cavity cell lines, Cal27 and Cal33, which were not responsive to EGF‐mediated EMT, FaDu, and Kyse30 cell lines, were characterized by low pEMT‐Singscores and low SLUG expression levels, thus providing a wider window of measurement (Fig. 4C and Fig. S4A).

**Fig. 5:**
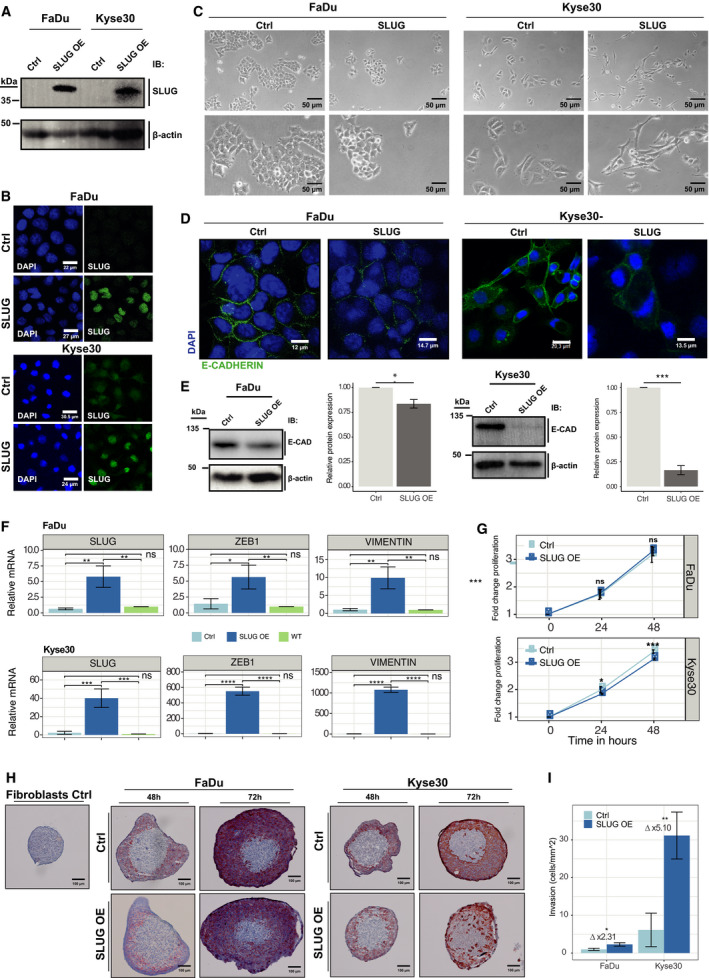
Exogenous SLUG expression induces phenotypic and functional characteristics of pEMT. (A) SLUG was overexpressed (SLUG OE) in FaDu and Kyse30 cells. SLUG expression levels of SLUG OE were assessed and compared with empty vector control cells (Ctrl) by immunoblotting with specific antibodies. (B) Expression and localization of SLUG were visualized by immunofluorescence laser scanning microscopy in control (Ctrl) and SLUG OE FaDu and Kyse30 cell lines (green). Nucleic DNA is visualized with DAPI (blue). Shown are representative examples from *n* = 3 independent experiments. Scale bars represent 22 µm 24 µm (FaDu), and 30.5 µm and 27 µm (Kyse30). (C) Cell morphology after SLUG overexpression was assessed by 40× and 80× magnified light microscopy of vector Ctrl and SLUG OE cells. Shown are representative examples from *n* = 3 independent experiments. Scale bars represent 50 µm. (D, E) Expression of E‐cadherin (green) in Ctrl and SLUG OE FaDu, and Kyse30 cell lines was visualized by immunofluorescence laser scanning microscopy (D) and quantified by western blotting of whole‐cell lysates (E, left panel). (E, right panel) Shown is a quantification of E‐cadherin protein amounts of SLUG OE vs. Ctrl cells from *n* = 3 independent experiments as mean with SD. Student’s *t*‐test. Scale bars in (D) represent 12, 14.7, 20.3, and 13.5 µm. (F) mRNA expression levels of SLUG, vimentin, and ZEB1 in Ctrl, SLUG OE, and wild‐type (WT) FaDu and Kyse30 cell lines are shown as mean with standard deviations from *n* = 3 independent experiments performed in triplicates. One‐way ANOVA *post hoc* Tukey HSD. (G) Cell proliferation of Ctrl and SLUG OE FaDu and Kyse30 cell lines over 48 h is shown as mean from *n* = 3 independent experiments with *n* = 6 replicates each. One‐way ANOVA *post hoc* Tukey HSD. Ns, not significant; *P‐value ≤ 0.05; **P‐value < 0.01; ***P‐value < 0.001; ****P‐value < 0.0001. (H) Normal human skin fibroblast spheroids were formed and co‐cultured with vector control (Ctrl) or SLUG overexpressing (SLUG OE) FaDu and Kyse30 cell lines. Fibroblast spheroid invasion after 48 and 72 h was visualized by pan‐cytokeratine IHC staining of cryosections. Shown are representative examples of *n* = 3 independent experiments with multiple spheroids. Scale bars represent 100 µm. (I) Invasion was quantified by Matrigel invasion assay. SLUG OE and Ctrl FaDu and Kyse30 cell lines were assessed after 24 h of invasion by cell counting. Shown are mean and SD of *n* = 3 independent experiments. Student’s t‐test. *P*‐value: *< 0.05; **< 0.01.

SLUG was overexpressed (SLUG‐OE) and localized in the nucleus in FaDu and Kyse30 cells (Fig. 5A,B). FaDu‐SLUG‐OE cells retained a primarily epithelial phenotype, however, with reduced cell–cell junctions, as judged from light microscopy phase‐contrast micrographs. Kyse30‐SLUG‐OE cells displayed morphological features of pEMT with reduced cell–cell contact and spindle‐shaped morphology (Fig. 5C). Loss of cell–cell contact was corroborated by reduced E‐cadherin levels in FaDu‐SLUG‐OE (16.3% reduction) and Kyse30‐SLUG‐OE cells (83.4% reduction) (Fig. 5D,E). FaDu and Kyse30 SLUG‐OE cells expressed similarly enhanced SLUG mRNA levels as compared to the respective controls (55‐ and 40‐fold, respectively). SLUG expression resulted in significantly higher levels of Zeb1 and vimentin mRNA, but the magnitude of Zeb1 and vimentin increase was one and two orders of magnitude higher in Kyse30 compared with FaDu cells, respectively (Fig. 5F). Proliferation rates of FaDu‐SLUG‐OE cells remained unchanged, whereas Kyse30‐SLUG‐OE cells displayed a significant but minor proliferation decrease (9.0% after 48 h) (Fig. 5G). SLUG overexpression moderately increased the transcription of five out of six top pEMT genes in FaDu, namely ITGA5, LAMC2, PDPN, TFGB1, and vimentin (Fig. S4, Fig. 5F). In Kyse30 cells, mRNA levels of three out of six top pEMT genes, namely ITGA5, TGFB1, and vimentin, were moderately or strongly increased. MMP10 was substantially decreased in SLUG‐OE cells for unknown reasons (Fig. S4, Fig. 5F).

Invasion of SLUG‐OE cells was addressed in a 3D co‐culture model in which control‐ or SLUG‐OE‐FaDu and ‐Kyse30 cells were added as single‐cell suspensions to preformed human fibroblasts spheroids (Fig. S5A). FaDu and Kyse30 control cell lines (cytokeratin‐staining) accumulated around fibroblast spheroids with no obvious signs of invasion (Fig. 5H, upper middle and right panels). FaDu‐SLUG‐OE cells showed moderately increased invasion of single and small aggregates into fibroblast spheroids (Fig. 5H, lower middle panels). Kyse30‐SLUG‐OE cells displayed a strong invasive phenotype with numerous invaded cells in the inner area of fibroblast spheroids (Fig. 5H, lower right panels). In a Matrigel invasion assay, FaDu‐SLUG‐OE cells showed a 2.31‐fold higher invasion over controls (2.29 ± 0.45 cell·mm^−2^ and 0.99 ± 0.28 cells·mm^−2^, respectively) (Fig. 5I, Fig. S5B). Parental Kyse30 cells revealed a 6.19‐fold higher invasive potential over FaDu cells. SLUG‐OE further fostered invasion by 5.10‐fold compared with Kyse30 controls (31.17 ± 6.23 cells·mm^−2^ and 6.13 ± 4.45 cells·mm^−2^, respectively) (Fig. 5I, Fig. S5B).

SLUG expression had no effect *per se* on percentages of alive, dead, and apoptotic cells (Fig. S6A). Proportions of living cells following induction of apoptosis with 100 and 500 nm of staurosporine were significantly higher in FaDu‐SLUG‐OE compared with controls, whereas the apoptotic fraction was significantly lower compared with controls (Fig. S6A). In Kyse30 cells, exposure to 500 nm of staurosporine resulted in significantly higher fraction of living SLUG‐OE cells (16.5% difference). 24.9% of Kyse30‐SLUG‐OE cells appeared in the dead cell fraction vs. 45.9% in controls (Fig. S6A).

Irradiation of FaDu control and SLUG‐OE cells with 10 Gy led to significant differences of alive fractions (Ctr.: 62.7% SLUG‐OE: 80.5%) and apoptotic fractions (Ctrl.: 27.3%, SLUG‐OE: 12.4%) (Fig. S6B). Kyse30 cells were generally more resistant to irradiation. When irradiated with 10 Gy, 85.3% of Kyse30 control cells and 92.8% of SLUG‐OE cells were contained in the alive fraction, and 13% of control cells and 2.8% of SLUG‐OE cells were present in the dead fraction (Fig. S6B). Upon fractionated irradiation of five times 2 Gy, 57.7% of FaDu control cells were alive and 32.6% dead. In contrast, 72.9% of FaDu‐SLUG‐OE cells were alive and 19.6% dead (Fig. S6C). Clonogenic assays of irradiated cells confirmed an enhanced resistance of FaDu‐SLUG‐OE cells following 2, 4, and 6 Gy and enhanced resistance of Kyse30‐SLUG‐OE cells following 4 Gy irradiation (Fig. S6D,E).

Taken together, SLUG promotes pEMT‐associated phenotypic and functional features including enhanced invasion and resistance to irradiation.

### 3.5 SLUG associates with recurrence and lymph node metastases

**Fig. 6:**
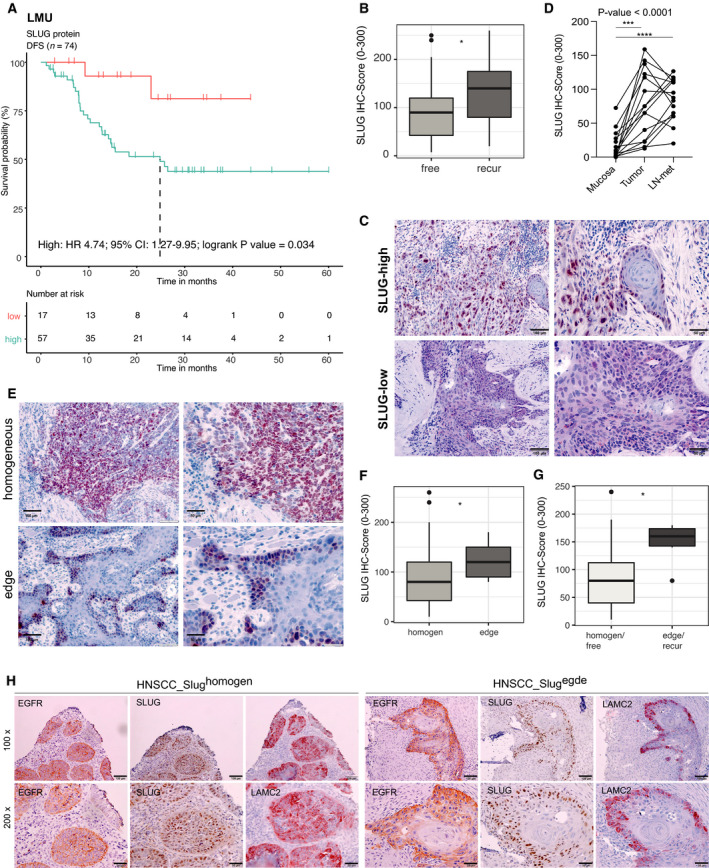
SLUG protein level is prognostic and associated with tumor progression. (A) Multivariable CoxPH was modeled for patients of the LMU cohort of HPV‐negative HNSCC patients (*n* = 74). Kaplan–Meier survival curve and table with logrank *P*‐value, Cox HR, and 95% CI after stratification according to SLUG IHC score [1st quartile (low) vs. 2nd–4th quartiles (high)] are shown. (B) Box plots show respective SLUG IHC score of patients suffering from recurrence vs. recurrence‐free. Wilcoxon test is shown. *P‐value ≤ 0.05. (C) Representative examples of high and low nuclear IHC SLUG staining intensity in primary tumors from *n* = 15 matched triplets of normal mucosa, primary tumors, and lymph node metastases. Scale bars represent 100 µm (left panels) and 50 µm (right panels). (D) Line plots of nuclear SLUG IHC score in tumor‐free mucosa, tumor, and lymph node metastases from *n* = 15 HNSCC patients. One‐way ANOVA *post hoc* Tukey HSD is shown with overall *P*‐value. Adjusted *P*‐values of multiple tests: ***P‐value = 0.0003, ****P‐value < 0.0001. (E) Representative examples of homogeneous or edge SLUG staining in primary tumors from *n* = 15 matched triplets of normal mucosa, primary tumors, and lymph node metastases. Scale bars represent 100 µm (left panels) and 50 µm (right panels). (F) Box plots of SLUG IHC scores according to protein localization (homogen., homogeneous or edge). Wilcoxon test is shown. *P‐value ≤ 0.05. (G) Box plots of SLUG IHC scores in patients of the LMU cohort showing a homogeneous SLUG expression and no recurrence vs. a SLUG expression at the periphery (edge) and recurrences. Wilcoxon test is shown. *P‐value ≤ 0.05. (H) Representative examples of EGFR, SLUG, and LAMC2 expression in HNSCC with homogeneous (left panels; *n* = 5 tumors) and edge expression of SLUG (right panels; *n* = 5 tumors) in 100× and 200× magnifications. Scale bars represent 100 µm.

Based on SLUG effects on invasion and increased resistance to irradiation *in vitro*, potential associations with recurrence and radiotherapy were assessed in HNSCC patients. SLUG protein was quantified in HPV‐negative HNSCC (LMU cohort; *n* = 76, Table S6) using IHC scoring [25]. Univariable CoxPH models were performed with *n* = 74 patients for whom all treatment information was available. Recurrence, irradiation, and SLUG protein expression were identified as significantly associated with DFS (Table S6). Multivariable CoxPH modeling (excluding recurrence as a univariable) confirmed irradiation and SLUG protein expression as independent prognostic markers (Fig. S7A). Kaplan–Meier curves were generated using SLUG protein expression as threshold (1st quartile). DFS was significantly decreased in SLUG^high^ patients compared with SLUG^low^ patients (Fig. 6A, HR 4.74, 95% CI: 1.27–9.95, logrank *P*‐value = 0.034). Further stratification of patients according to radiotherapy showed a strong tendency toward improved DFS for SLUG^low^ patients compared with SLUG^high^ patients (Fig. S7B, HR 0.296, 95% CI: 0.06834–1.282, logrank *P*‐value = 0.1035). Patients with any recurrence including local, locoregional, and distant tumors/metastases had significantly poorer OS (Fig. S7C; HR: 2.51, 95% CI: 1.02–6.21, *P* = 0.039) and expressed higher levels of SLUG protein in the index carcinoma compared with patients without any recurring tumor over five years of clinical follow‐up (Fig. 6B).

A panel of tumor‐free mucosa, primary tumor, and lymph node metastases was obtained from *n* = 15 HPV‐negative HNSCC patients (Table S7), and IHC scoring for SLUG expression was performed (see examples of SLUG‐high and SLUG‐low tumors in Fig. 6C). Nuclear SLUG expression was significantly increased in primary tumors (IHC score mean: 77.98/median: 75) and in lymph node metastases (IHC score mean: 83.33; median: 87.5) compared with tumor‐free mucosa (IHC score mean: 16.46/median: 7.5) (Fig. 6D).

Within the full cohort, SLUG expression patterns were either homogeneous or enriched at the tumor–stroma interface (edge) (Fig. 6E) with the latter being associated with higher overall expression (Fig. 6F). Recurrence‐free patients with a homogeneous SLUG expression were characterized by substantially lower SLUG expression than patients with recurrences and a localization of SLUG at the edges of tumor areas (Fig. 6G). Furthermore, SLUG colocalized with EGFR and pEMT marker LAMC2 in selected patients with a homogeneous or a peripheral SLUG expression at the edges of tumor areas (Fig. 6H). Hence, strong and preferably peripheral expression of SLUG in primary HNSCC correlated with tumor recurrence and disease progression.

## 4 Discussion

Proportions of malignant pEMT cells determined by scRNAseq in OSCC correlated with adverse pathologic parameters and disease progression [5] and pEMT gene signatures may represent novel molecular classifiers for disease prediction. However, large‐scale scRNAseq screens are not a worldwide standard‐of‐care. Our aim was to develop a quantification protocol for pEMT applicable to bulk sequencing and microarray data of large clinical cohorts of HNSCC, which are more easily available in clinical settings. pEMT was quantified using *n* = 15 common pEMT genes [5] by applying the cell type‐specific deconvolution algorithm EPIC [29] in combination with single‐sample scoring of molecular phenotypes (Singscoring) [22, 30]. EPIC allowed to determine proportions of nonmalignant cells in HNSCC cohorts and confirmed CAFs as major source of pEMT gene expression in nonmalignant cells that interferes with pEMT quantification in bulk sequencing (Fig. 1) [5]. Applying pEMT‐Singscores demonstrated that the common pEMT gene signature, but not multiple random 15 genes, is an independent molecular prognosticator of poor OS in three independent HNSCC cohorts and of reduced response to irradiation in TCGA (Fig. 2). Hence, reducing dimensional complexity of pEMT using Singscores proved to be feasible at the single patient level in deconvoluted bulk sequencing and microarray data.

High pEMT‐Singscores in the absence of nodal metastases correlated with similar survival probabilities than nodal metastases in the context of low pEMT in irradiated patients (Fig. 2H). We conclude that pEMT has a comparably important negative impact on disease control as the established parameter of nodal involvement. Prognostic pEMT‐Singscores were generated from primary tumors suggesting an influence of malignant pEMT cells present already at initial diagnosis. A selection and contribution of malignant pEMT cells during disease progression in developing treatment resistance are a likely scenario [8, 10, 31]. At the global transcriptional level, 34 of 100 pEMT genes that compose the pEMT signature in HNSCC were recovered as DEGs in pEMT^high^ patients compared with pEMT^low^ patients in all three cohorts analyzed. GO term analyses demonstrated that patients with high pEMT‐Singscores were characterized by gene expression profiles associated with cell motility and migration, which may support local dissemination, at the expense of metabolic activity and oxidative phosphorylation (Fig. 3). Hence, our results demonstrate a general impact of pEMT on disease progression, therapeutic response, and clinical outcome in HNSCC.

A high pEMT‐Singscore could represent a supportive rationale for aggressive treatment regimens, whereas pEMT low‐risk patients might profit from treatment de‐escalation, for example, with respect to irradiation and concurrent chemotherapy. However, clinical implementation of pEMT quantification, for example, using Singscores, requires further studies in prospective cohorts and the establishment of thresholds for risk evaluation. In fact, few molecular classifiers have found their way into clinical routine and have obtained approval by the FDA and/or the EMA, such as Oncotype DX and MammaPrint in breast cancer [32]. Hence, such clinical translation remains an important and demanding endeavor.

In search of regulators of pEMT in HNSCC, we could show that SLUG mRNA expression correlated best with pEMT‐Singscores and common pEMT genes in clinical cohorts and cell lines. All other EMT‐TFs were only poorly or not correlated to pEMT‐SingScores (Fig. 4). Based on published functions of SLUG in EMT regulation [18, 33], we suggest that SLUG is not only a surrogate for pEMT but rather actively contributes to induce a pEMT phenotype in HNSCC. This is supported by results showing that ectopic SLUG expression in cell lines of the upper aerodigestive tract induces functions such as invasion and decreased sensitivity to irradiation (Fig. 5), which are commonly attributed to pEMT. SLUG protein expression in an in‐house HNSCC cohort further highlighted its association with disease recurrence, poorer response to radiotherapy, and the presence of clusters of pEMT‐type malignant cells at the edge of tumor areas (Fig. 6 and Fig. S7), which is in accordance with reports from others [34, 35, 36]. A recent meta‐analysis of the prognostic value of EMT‐TFs in HNSCC disclosed that Twist, Snail, SLUG, and Zeb1 correlated with significantly poorer OS [37]. Further reports demonstrated SNAIL and TWIST as major inducers of EMT in HNSCC [38, 39, 40]. Despite clearly inferior Spearman correlations in all cohorts analyzed, it cannot be formally excluded that combinatorial functions of SLUG, SNAIL, TWIST1, and others orchestrate the complex pEMT phenotype. However, a meta‐analysis across breast cancer studies and a study across different cancer entities including HNSCC identified SLUG as main regulator of EMT [41, 42]. In this respect, it is noteworthy that SLUG was the only EMT‐TF in pEMT^high^‐associated DEGs in TCGA, MDACC, and FHCRC and likewise the only EMT‐TF associated with the pEMT signature in OSCC, although not at the single‐cell level [5].

Regulatory mechanisms of SLUG expression in HNSCC are not fully understood. Gao *et al*. [43] demonstrated that loss of E‐cadherin along with an increased expression of vimentin and SLUG at tumor margins was related to the activation of the EGFR/ERK‐pathway through the release of EGF by cancer‐associated macrophages in the stroma. In concordance with a role for the tumor microenvironment [44] and the EGFR pathway more specifically, our group reported that the strong activation of EGFR by EGF resulted in EMT induction with an up‐regulation of SNAIL, SLUG, and ZEB1 [23]. Accordingly, patients with a high expression of EGFR or of phosphorylated ERK and/or SLUG were characterized by poorer OS [23]. We propose that EGFR overexpression as observed in HNSCC [45] may have several negative repercussion on clinical outcome beyond established roles in tumor cell proliferation [45, 46, 47]. Enhanced EGFR signaling may promote a strong activation of ERK and pEMT via SLUG in HNSCC, preferentially at the tumor margin. As a result, pEMT might confer an increased invasive potential to SLUG‐positive carcinoma cells allowing them to locally invade and generate deposits of tumor with enhanced therapy resistance that are the seeds of minimal residual disease and future recurrences. Strong EGFR signaling and ERK2 expression were associated with immune evasion in HNSCC [48, 49] and ERK2 was correlated with recurrences [49]. Hence, inhibition of EGFR and more specifically of MEK1/2 pathways might reveal potent pEMT blockade in combination with radiotherapy and in therapy sensitization [50]. Both, *in vitro* data from the present study on the enhanced invasive potential of SLUG‐positive carcinoma cells and improved resistance to irradiation, and positive correlations of SLUG *in vivo* with recurrences, in particular local recurrences and with EGFR and the pEMT marker LAMC2, are in support of this notion (Fig. 6).

## 5 Conclusions

In conclusion, pEMT is a main parameter of tumor progression that can be quantified using cell type‐specific deconvolution and Singscores, and which strongly correlates with SLUG expression in HNSCC. Prognostic pEMT‐Singscores could find application in the clinical setting to stratify patients more precisely into subgroups with differing risk of disease spread, recurrence, and therapy response. Patients at high risk would profit from a longitudinal monitoring and could, potentially, benefit from more aggressive treatments to suppress tumor recurrence based on a pEMT‐related biomarker‐driven stratification. Furthermore, high pEMT‐Singscores may provide a rationale for the addition of EGFR‐targeted therapies beyond palliative treatment conditions.

## Conflict of interest

The authors declare no conflict of interest.

## Author contributions

HS and OG contributed to conceptualization; OG, HS, and PB contributed to methodology; HS involved in software utilization; HS, OG, and PB made validation; HS, MA, MP, TQ, and JZ made formal analysis; HS, MA, ES, and OG investigated the study; GK, DL, PB, OG, MC, FS, and HS contributed to resources; HS, MA, JZ, FS, and PB curated the data; HS involved in writing original draft preparation; OG, MC, and PB involved in writing review and editing; HS, OG, and MA contributed to visualization; OG and PB supervised the study; OG and PB contributed to project administration; and OG and FS acquired funding.

### Peer Review

The peer review history for this article is available at https://publons.com/publon/10.1002/1878‐0261.13075.

## Supporting information


**Fig. S1.** pEMT‐Singscores correlation with nonmalignant cell types in HNSCC. pEMT‐Singscores were calculated for n = 15 common pEMT genes for patients in the TCGA (A), MDACC (B), and FHCRC (C) cohorts following cohort deconvolution using the EPIC algorithm. Correlations of pEMT‐Singscores with the indicated nonmalignant cell types are shown with Spearman’s rho and p‐values.

**Fig. S2.** Computation of pEMT‐Singscores based on n = 15 common pEMT genes in TCGA cohort. (A) Upper left: Quantile–quantile plot of pEMT‐Singscores of common pEMT genes vs. theoretical quantiles within the TCGA HNSCC cohort. Lower: Rank density plot of pEMT‐Singscores from common pEMT genes shows patients with lowest (right) and patient with highest pEMT‐Singscore (left) in normalized gene ranks. Upper right: Dot plot of dispersion against common pEMT‐Singscores of each TCGA patient. (B, C) Same as (A) for patients of the MDACC and FHCRC cohorts. (D) pEMT‐Singscores were calculated for n = 15 common pEMT genes for patients in the TCGA cohort who received therapeutic irradiation (n = 133) and served to compute a multivariable Cox proportional hazard model. Shown is a Forest plot including all univariables significantly associated with OS in a multivariable Cox proportional hazard model with patient numbers, linear hazard ratio, 95% CI, logrank p‐value, AIC, and concordance indexes.

**Fig. S3.** Correlation of pEMT‐Singscores with epithelial and mesenchymal markers in ESCC and HNSCC cell lines. pEMT‐Singscores were computed in ESCC and HNSCC cell lines of the CCLE database and are plotted against expression values of epithelial markers E‐cadherin (CDH1), EGFR, EpCAM, and mesenchymal markers N‐cadherin (CDH2), Fibronectin 1 (FN1), and vimentin (VIM). Esophageal (ESCC) and HNSCC cell lines are depicted as red and blue dots, respectively. Spearman’s rho and p‐values are indicated.

**Fig. S4.** Top pEMT genes and SLUG overexpression. RT‐qPCR mRNA quantification of (A) SLUG expression in Kyse30, FaDu, Cal27, and Cal33 and (B) top pEMT genes ITGA5, LAMC2, MMP10, PDPN, and TGFB1 of vector control (Ctrl) and SLUG overexpressing cells (SLUG OE) FaDu and Kyse30 cell lines. Normalized to vector control cells (Ctrl). Student’s t‐test. Ns: not significant; * p‐value ≤ 0.05; ** p‐value < 0.01; ***.

**Fig. S5.** Spheroid invasion and transmigration. (A) Light microscopic images with 10x magnification of normal human skin fibroblasts spheroids (left) and co‐cultured SLUG overexpressing (SLUG OE) and vector control (Ctrl) FaDu and Kyse30 cells (middle and right) after 72 hours. Scale bars represent 305 µm. (B) Light microscopic images with 40x magnification of SLUG OE and Ctrl FaDu and Kyse30 cells invaded onto the bottom of membranes from Matrigel invasion assays after 24 hours. Cells were fixed and stained with crystal violet. Shown are representative images of n = 3 independent experiments. Scale bars represent 1231 µm.

**Fig. S6.** Staurosporine treatment and irradiation of control and SLUG‐OE cell lines. (A) SLUG OE and Ctrl FaDu and Kyse30 cell lines were treated for 24 hours with 100 and 500 nM of Staurosporine. Cell death was assessed by flow cytometry and Annexin V/PI staining. Shown are mean and standard deviations of n = 3 independent experiments. (B‐C) SLUG OE and Ctrl FaDu and Kyse30 cell lines were irradiated with 10 and 2 Gray (Gy) and after 72 hours cell death was assessed by Annexin V/PI staining. 10 Gy were also applied as fractionation in 5x 2 Gy shown in (C). Shown are mean and standard deviations of n = 3 independent experiments. (D) Clonogenic survival assay of SLUG OE vs. Ctrl FaDu and Kyse30 cell lines with 0 (Control), 2, 4, and 6 Gy irradiation. Area of colonies was measured by ColonyArea ImageJ Plugin after 2 weeks for FaDu and 10 days for Kyse30 cells. Shown are mean and standard deviations of n = 3 independent experiments. (C‐F) one‐way ANOVA post hoc Tukey HSD. Ns—not significant; * p‐value ≤ 0.05; ** p‐value < 0.01; ***. (E) BW images of 6‐well plates after 14 days (FaDu) or 10 days (Kyse30) of SLUG OE and Ctrl cells with different doses of irradiation as stated. Shown are representative results from n = 3 independent experiments.

**Fig. S7.** SLUG protein expression and correlations with clinical endpoints. (A) Univariable served to compute a multivariable Cox proportional hazard model. Shown is a Forest plot including all univariables significantly associated with OS in a multivariable Cox proportional hazard model with patient numbers, linear hazard ratio, 95% CI, logrank p‐value, AIC, and concordance indexes. (B) Multivariable CoxPH was modeled for patients of the LMU cohort of HPV‐negative HNSCC patients (n = 74; logrank p‐value 0.014). Kaplan–Meier survival curve and table with logrank p‐value, Cox HR, and 95% CI after stratification according to SLUG IHC score (1^st^ quartile (low) vs. 2^nd^‐4^th^ quartiles (high)) and radiation status are shown. (C) Kaplan–Meier survival curve and table showing the overall survival (OS) based on the occurrence of tumor recurrences.

**Table S1.** STR typing results of FaDu and Kyse‐30 cell lines.

**Table S2.** Clinical parameters of the TCGA HNSCC cohort implemented in uni‐ and multivariable analyses.

**Table S3.** Clinical parameters of the MDACC HNSCC cohort implemented in uni‐ and multivariable analyses.

**Table S4.** Clinical parameters of the FHCRC OSCC cohort implemented in uni‐ and multivariable analyses.

**Table S5.** Gene lists of up‐ and down‐regulated genes in the TCGA, MDACC and FHCRC cohorts identified using DESeq, edgeR and limma/limma‐voom algorithms and pEMT‐Singscore^low^ patients (lower 40%) as baseline to compare with pEMT‐Singscore^high^ patients (upper 40%).

**Table S6.** Clinical parameters of the LMU HNSCC cohort implemented in uni‐ and multivariable analyses.

**Table S7.** Clinical parameters of the matched triplets of normal mucosa, primary tumor and lymph node metastases analyzed for Slug expression.

## Data Availability

Datasets generated during and/or analyzed during the current study are either publicly available [TCGA [2], MDACC (GEO object GSE42743), and FHCRC (GEO object GSE41613)] or are available from the corresponding author on reasonable request. All codes and r‐packages used in the study are publicly available and have been disclosed in Methods or are available from the corresponding author on reasonable request.
