# Supplementary figures and images for: SLUG‐related partial epithelial‐to‐mesenchymal transition is a transcriptomic prognosticator of head and neck cancer survival
Source: Mol Oncol. 2021 Aug 21;16(2):347–67. doi: 10.1002/1878-0261.13075 (PMC8763659; doi:10.1002/1878-0261.13075)

**A**

**TCGA**

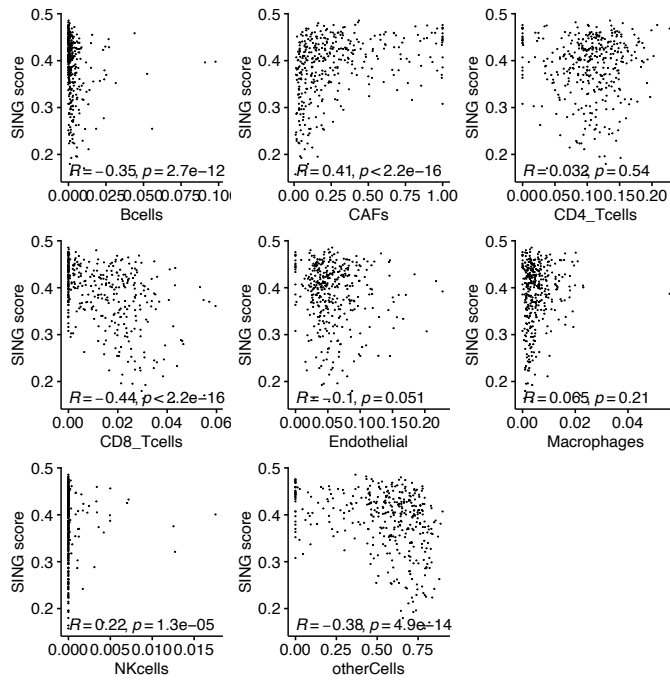

**B**

**MDACC**

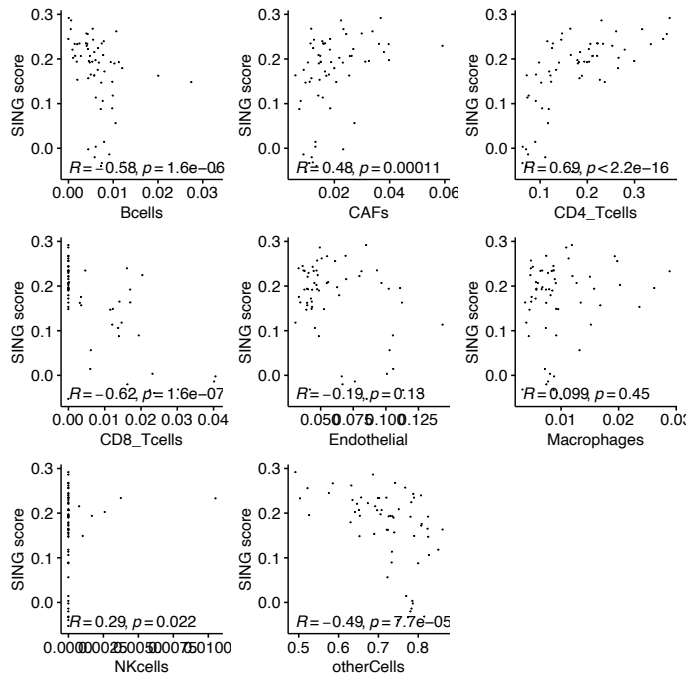

**C**

**FHCRC**

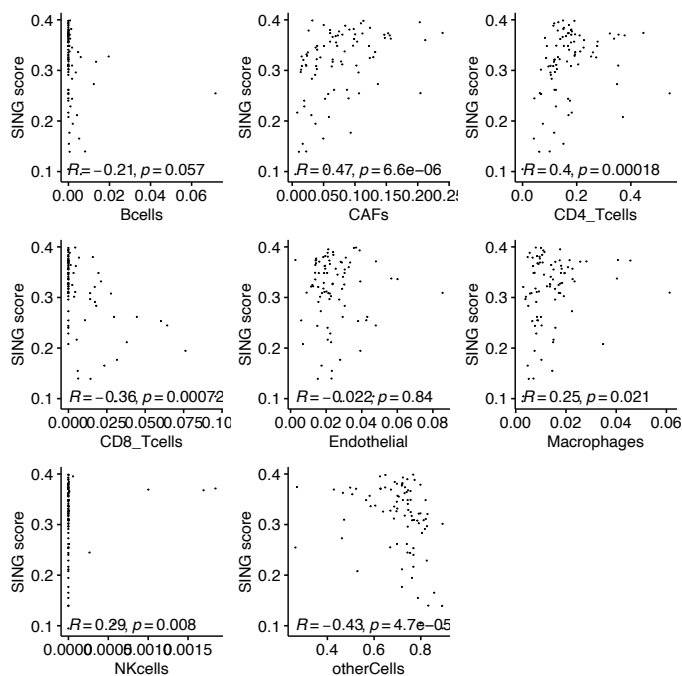

Supplement: Supplementary file 1 — Fig. S1. pEMT‐Singscores correlation with nonmalignant cell types in HNSCC. pEMT‐Singscores were calculated for n = 15 common pEMT genes for patients in the TCGA (A), MDACC (B), and FHCRC (C) cohorts following cohort deconvolution using the EPIC algorithm. Correlations of pEMT‐Singscores with the indicated nonmalignant cell types are shown with Spearman’s rho and p‐values. [file MOL2-16-347-s014.pdf]

## A TCGA

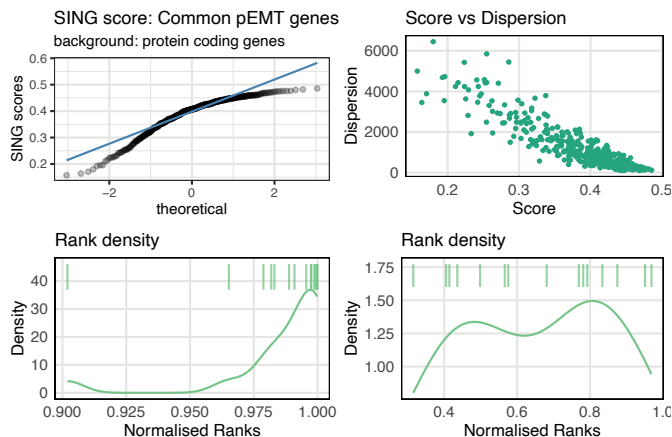

## B MDACC

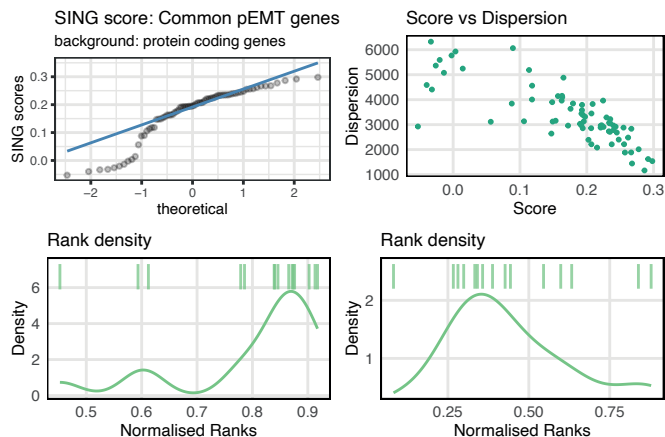

## C FHCRC

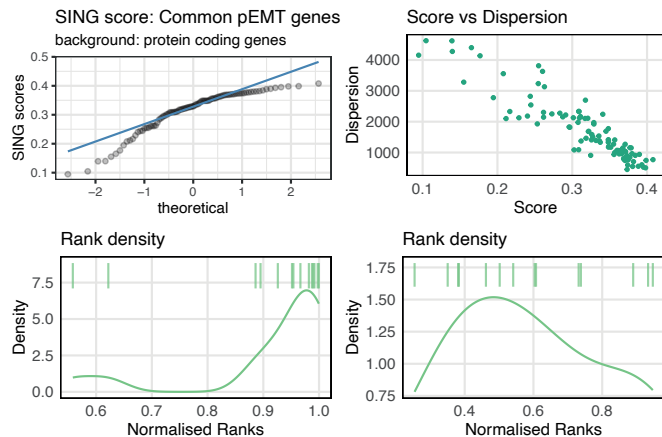

## D TCGA

### RT patients (n = 133)

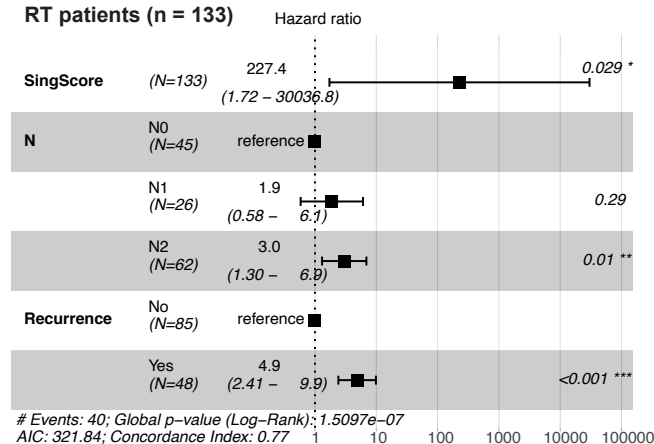

Supplement: Supplementary file 2 — Fig. S2. Computation of pEMT‐Singscores based on n = 15 common pEMT genes in TCGA cohort. (A) Upper left: Quantile–quantile plot of pEMT‐Singscores of common pEMT genes vs. theoretical quantiles within the TCGA HNSCC cohort. Lower: Rank density plot of pEMT‐Singscores from common pEMT genes shows patients with lowest (right) and patient with highest pEMT‐Singscore (left) in normalized gene ranks. Upper right: Dot plot of dispersion against common pEMT‐Singscores of each TCGA patient. (B, C) Same as (A) for patients of the MDACC and FHCRC cohorts. (D) pEMT‐Singscores were calculated for n = 15 common pEMT genes for patients in the TCGA cohort who received therapeutic irradiation (n = 133) and served to compute a multivariable Cox proportional hazard model. Shown is a Forest plot including all univariables significantly associated with OS in a multivariable Cox proportional hazard model with patient numbers, linear hazard ratio, 95% CI, logrank p‐value, AIC, and concordance indexes. [file MOL2-16-347-s010.pdf]

# Epithelial marker

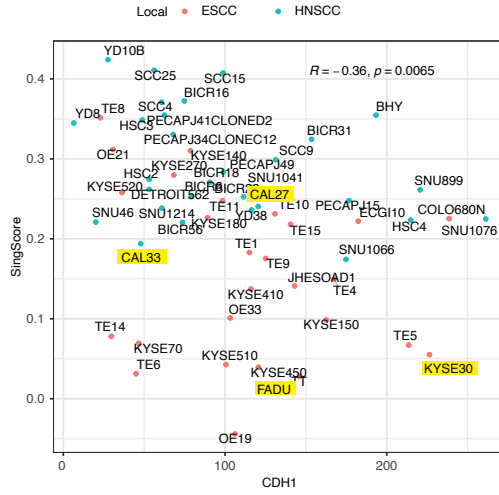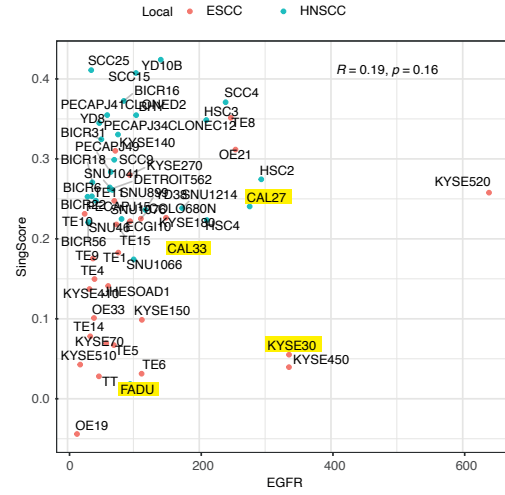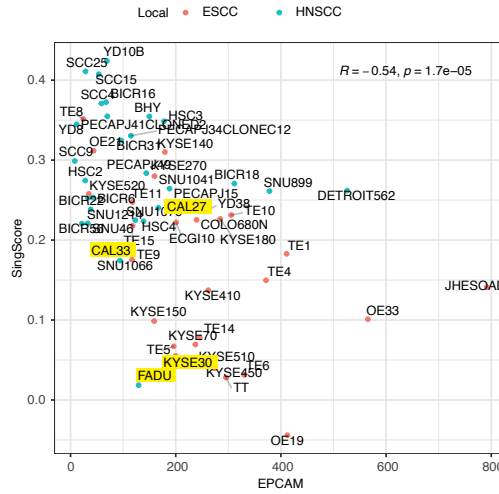

# Mesenchymal marker

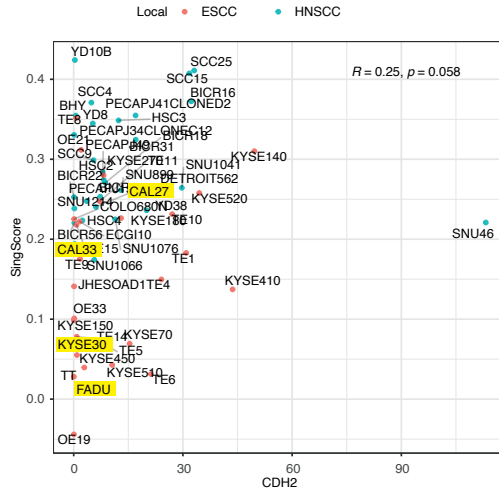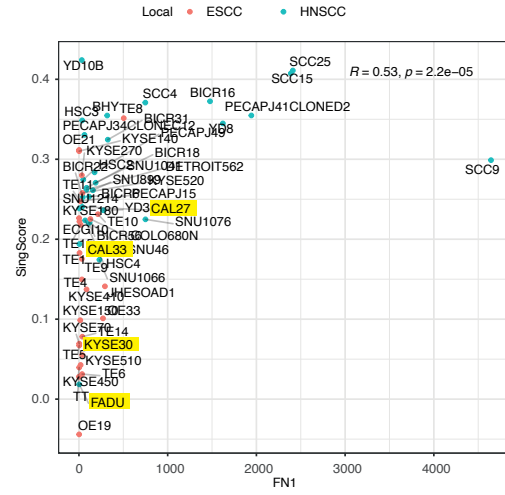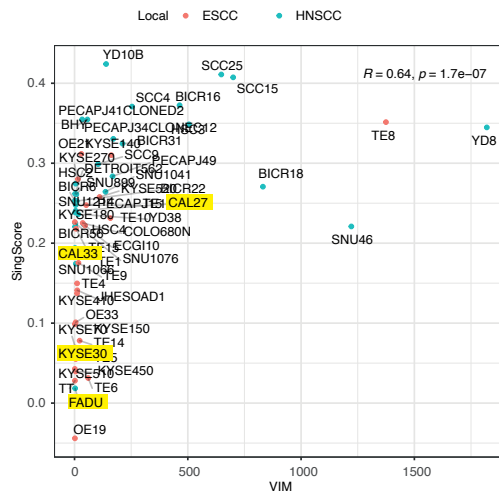

Supplement: Supplementary file 3 — Fig. S3. Correlation of pEMT‐Singscores with epithelial and mesenchymal markers in ESCC and HNSCC cell lines. pEMT‐Singscores were computed in ESCC and HNSCC cell lines of the CCLE database and are plotted against expression values of epithelial markers E‐cadherin (CDH1), EGFR, EpCAM, and mesenchymal markers N‐cadherin (CDH2), Fibronectin 1 (FN1), and vimentin (VIM). Esophageal (ESCC) and HNSCC cell lines are depicted as red and blue dots, respectively. Spearman’s rho and p‐values are indicated. [file MOL2-16-347-s001.pdf]

**A**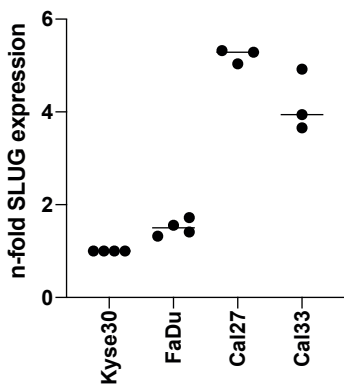**B**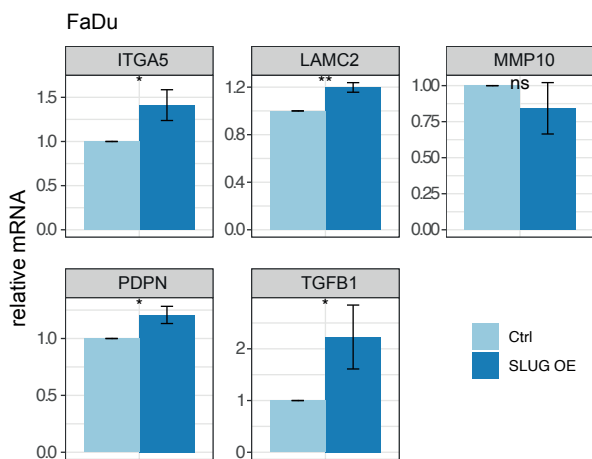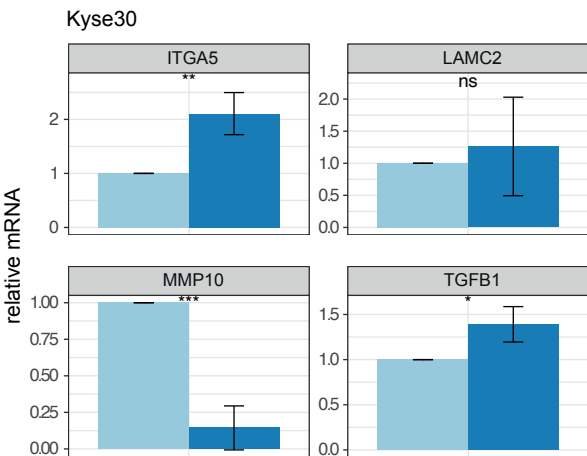

Supplement: Supplementary file 4 — Fig. S4. Top pEMT genes and SLUG overexpression. RT‐qPCR mRNA quantification of (A) SLUG expression in Kyse30, FaDu, Cal27, and Cal33 and (B) top pEMT genes ITGA5, LAMC2, MMP10, PDPN, and TGFB1 of vector control (Ctrl) and SLUG overexpressing cells (SLUG OE) FaDu and Kyse30 cell lines. Normalized to vector control cells (Ctrl). Student’s t‐test. Ns: not significant; * p‐value ≤ 0.05; ** p‐value < 0.01; ***. [file MOL2-16-347-s005.pdf]

**A**Fibroblasts onlyFaDuKyse30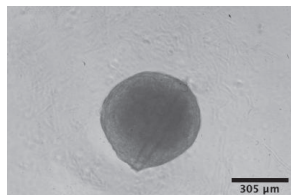

Ctrl

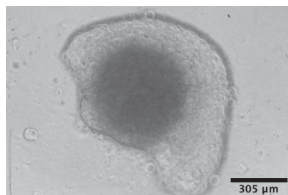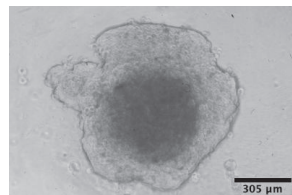

SLUG OE

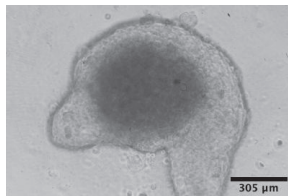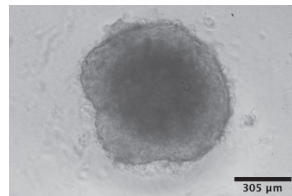**B**FaDuKyse30

Ctrl

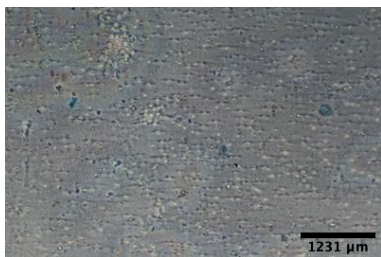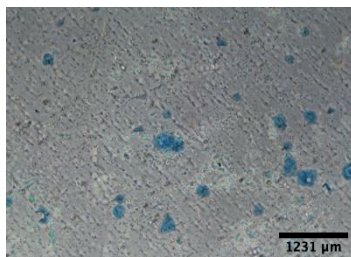

SLUG OE

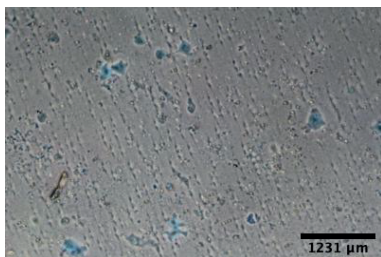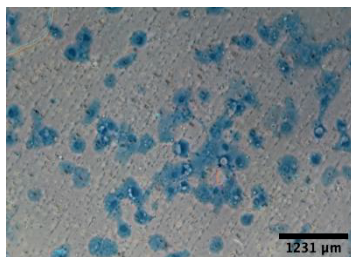

Supplement: Supplementary file 5 — Fig. S5. Spheroid invasion and transmigration. (A) Light microscopic images with 10x magnification of normal human skin fibroblasts spheroids (left) and co‐cultured SLUG overexpressing (SLUG OE) and vector control (Ctrl) FaDu and Kyse30 cells (middle and right) after 72 hours. Scale bars represent 305 µm. (B) Light microscopic images with 40x magnification of SLUG OE and Ctrl FaDu and Kyse30 cells invaded onto the bottom of membranes from Matrigel invasion assays after 24 hours. Cells were fixed and stained with crystal violet. Shown are representative images of n = 3 independent experiments. Scale bars represent 1231 µm. [file MOL2-16-347-s007.pdf]

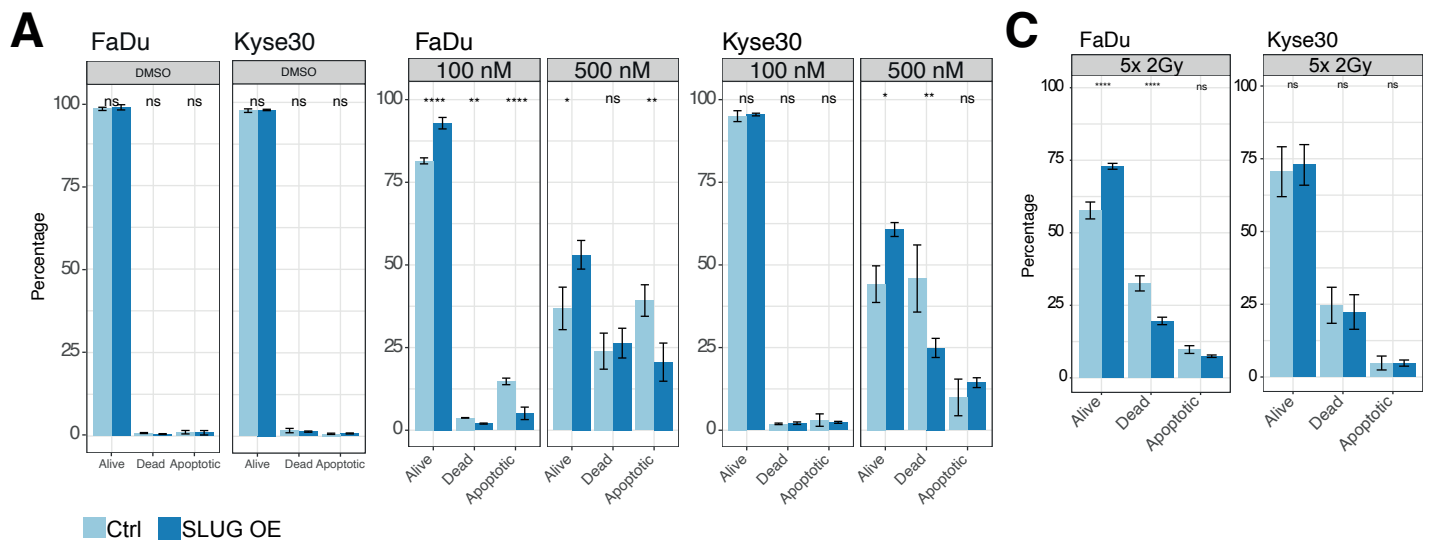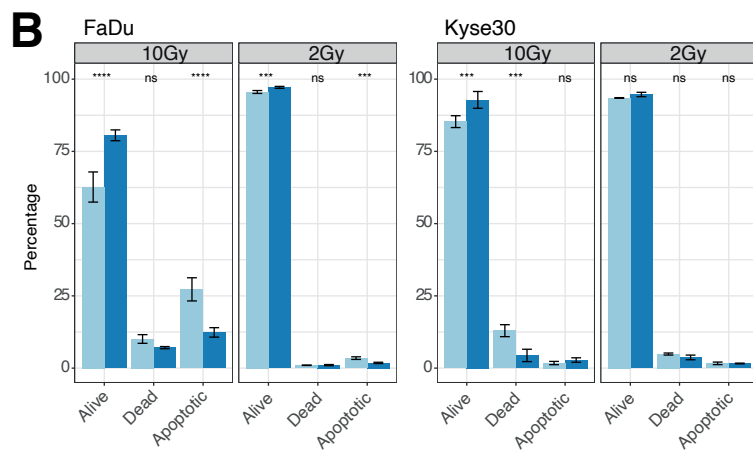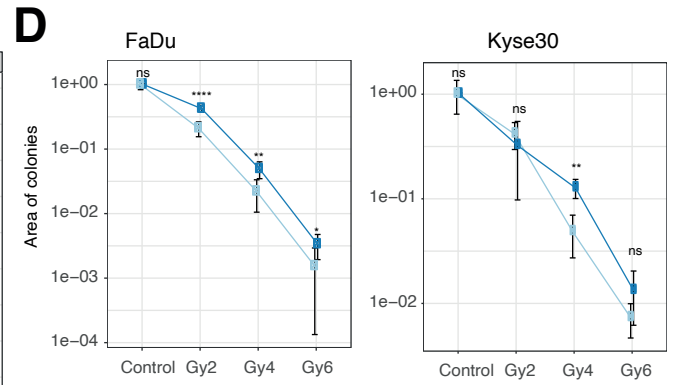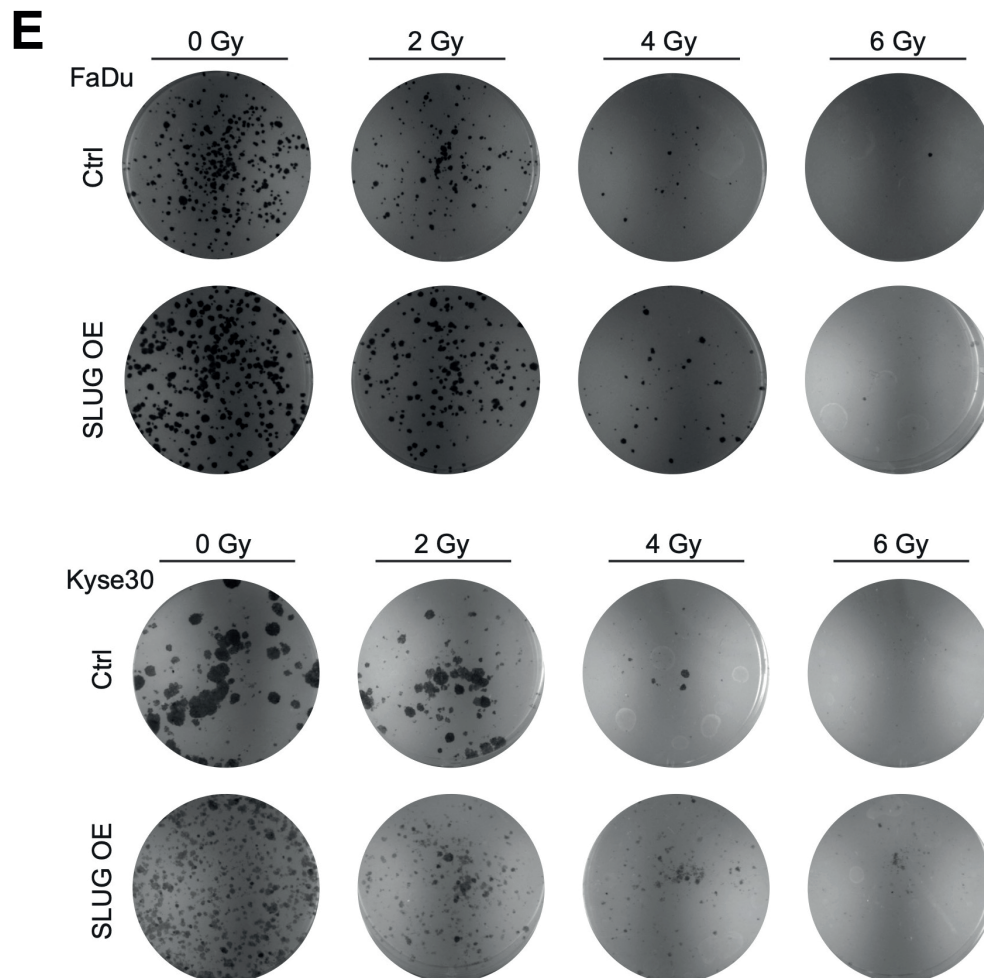

Supplement: Supplementary file 6 — Fig. S6. Staurosporine treatment and irradiation of control and SLUG‐OE cell lines. (A) SLUG OE and Ctrl FaDu and Kyse30 cell lines were treated for 24 hours with 100 and 500 nM of Staurosporine. Cell death was assessed by flow cytometry and Annexin V/PI staining. Shown are mean and standard deviations of n = 3 independent experiments. (B‐C) SLUG OE and Ctrl FaDu and Kyse30 cell lines were irradiated with 10 and 2 Gray (Gy) and after 72 hours cell death was assessed by Annexin V/PI staining. 10 Gy were also applied as fractionation in 5x 2 Gy shown in (C). Shown are mean and standard deviations of n = 3 independent experiments. (D) Clonogenic survival assay of SLUG OE vs. Ctrl FaDu and Kyse30 cell lines with 0 (Control), 2, 4, and 6 Gy irradiation. Area of colonies was measured by ColonyArea ImageJ Plugin after 2 weeks for FaDu and 10 days for Kyse30 cells. Shown are mean and standard deviations of n = 3 independent experiments. (C‐F) one‐way ANOVA post hoc Tukey HSD. Ns—not significant; * p‐value ≤ 0.05; ** p‐value < 0.01; ***. (E) BW images of 6‐well plates after 14 days (FaDu) or 10 days (Kyse30) of SLUG OE and Ctrl cells with different doses of irradiation as stated. Shown are representative results from n = 3 independent experiments. [file MOL2-16-347-s004.pdf]

**A**

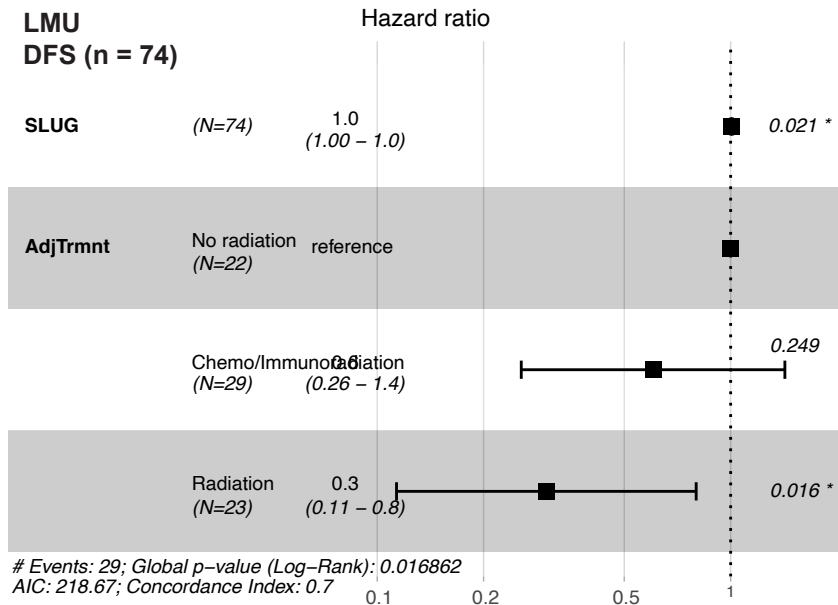

**B**

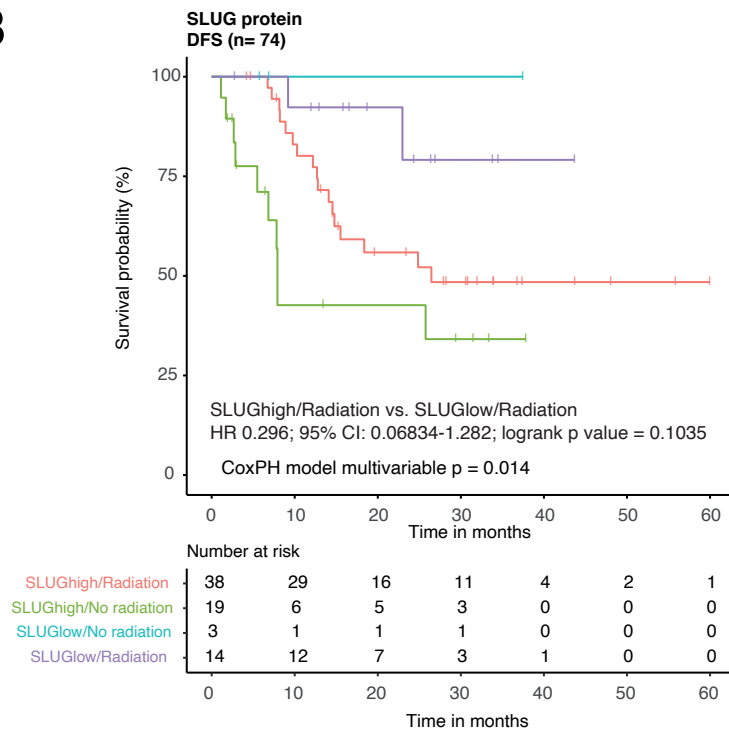

**C**

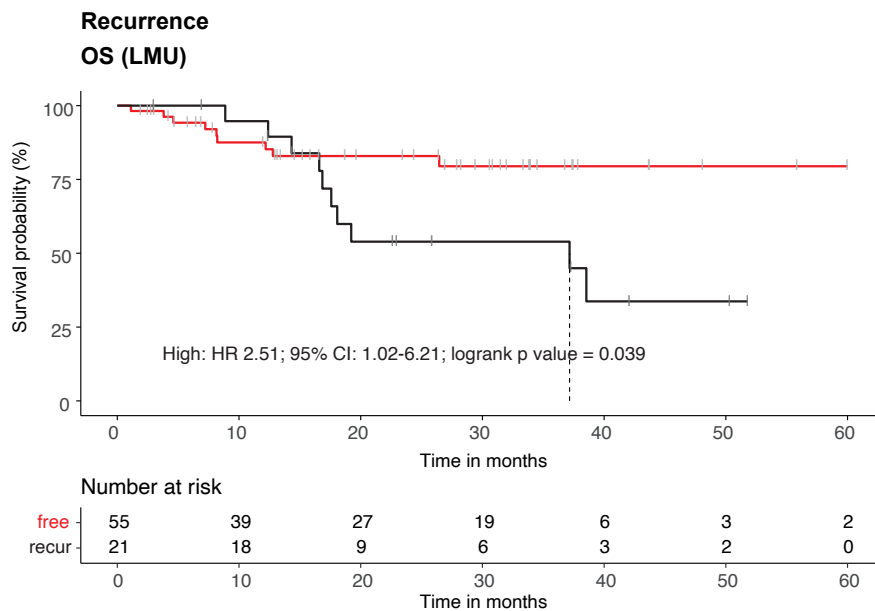

Supplement: Supplementary file 7 — Fig. S7. SLUG protein expression and correlations with clinical endpoints. (A) Univariable served to compute a multivariable Cox proportional hazard model. Shown is a Forest plot including all univariables significantly associated with OS in a multivariable Cox proportional hazard model with patient numbers, linear hazard ratio, 95% CI, logrank p‐value, AIC, and concordance indexes. (B) Multivariable CoxPH was modeled for patients of the LMU cohort of HPV‐negative HNSCC patients (n = 74; logrank p‐value 0.014). Kaplan–Meier survival curve and table with logrank p‐value, Cox HR, and 95% CI after stratification according to SLUG IHC score (1st quartile (low) vs. 2nd‐4th quartiles (high)) and radiation status are shown. (C) Kaplan–Meier survival curve and table showing the overall survival (OS) based on the occurrence of tumor recurrences. [file MOL2-16-347-s012.pdf]
