# Supplementary material for: SLUG‐related partial epithelial‐to‐mesenchymal transition is a transcriptomic prognosticator of head and neck cancer survival
Source: Mol Oncol. 2021 Aug 21;16(2):347–67. doi: 10.1002/1878-0261.13075 (PMC8763659; doi:10.1002/1878-0261.13075)
Supplement: Supplementary file 9 — Table S2. Clinical parameters of the TCGA HNSCC cohort implemented in uni‐ and multivariable analyses. [file MOL2-16-347-s013.docx]

**Supplementary Table 2:** Clinical parameters of the TCGA HNSCC cohort implemented in uni- and multivariable analyses. n.d.: not defined. OS: overall survival; DFS: Disease-free survival; PFS: Progression-free survival; DSS: Disease-specific survival; UICC: Union for International Cancer Control, HR: Hazard ratio; 95% CI: 95% confidence interval. References for categorical variables are indicated. Significant p-values are indicated: * < 0.05; ** < 0.01; *** < 0.001.

| **Number of patients** | n = 220 |
| --- | --- |
| **OS (months)** |  |
| Median | 20.630 |
| Mean | 25.297 |
| Range | 0.066 - 60 |
| **DFS (months)** |  |
| Median | 23.342 |
| Mean | 26.055 |
| Range | 4.8 - 60 |
| **PFS (months)** |  |
| Median | 15.616 |
| Mean | 22.004 |
| Range | 0.066 - 60.000 |
| **DSS (months)** |  |
| Median | 20.630 |
| Mean | 25.297 |
| Range | 0.066 - 60 |
| **HPV status** |  |
| HPV- | 220 (100.0%) |
| **Age (years)** |  |
| Median | 61 |
| Mean | 60.868 |
| Range | 24 - 90 |
| HR 1.01; 95% CI 0.991-1.03; p-value = 0.276 (OS) |  |
| **Primary sites** |  |
| Alveolar ridge (reference) | 11 (5.0%) |
| Base of tongue | 7 (3.2%) |
| HR 2.02; 95% CI 0.502-8.13; p-value = 0.323 (OS) |  |
| Buccal mucosa | 13 (5.9%) |
| HR 1.47; 95% CI 0.415-5.22; p-value = 0.55 (OS) |  |
| Floor of mouth | 32 (14.5%) |
| HR 1.48; 95% CI 0.485-4.49; p-value = 0.493 (OS) |  |
| Hard palate | 11 (5.0%) |
| HR 2.39; 95% CI 0.265-21.6; p-value = 0.437 (OS) |  |
| Hypopharynx | 4 (1.8%) |
| HR 4.06; 95% CI 0.735-22.4; p-value = 0.108 (OS) |  |
| Larynx | 56 (25.5%) |
| HR 0.577; 95% CI 0.187-1.78; p-value = 0.337 (OS) |  |
| Lip | 2 (0.9%) |
| (OS)HR 1.04e-07; 95% CI 0-inf; p-value = 0.997 |  |
| Oral cavity | 23 (10.5%) |
| HR 1.69; 95% CI 0.539-5.32; p-value = 0.367 (OS) |  |
| Oral tongue | 64 (29.1%) |
| HR 1.06; 95% CI 0.363-3.11; p-value = 0.0.912 (OS) |  |
| Oropharynx | 3 (1.4%) |
| HR 1.04e-07; 95% CI 0-inf; p-value = 0.996 (OS) |  |
| Tonsil | 3 (1.4%) |
| HR 2.08; 95% CI 0.38-11.4; p-value = 0.399 (OS) |  |
| **Stage (UICC)** |  |
| I (reference) | 13 (5.9%) |
| II | 29 (13.2%) |
| HR 0.621; 95% CI 0.113-3.41; p-value = 0.584 (OS) |  |
| III | 45 (20.5%) |
| HR 1.85; 95% CI 0.418-8.14; p-value = 0.418 (OS) |  |
| IV | 133 (60.5%) |
| HR 2.89; 95% CI 0.705-11.9; p-value = 0.14 (OS) |  |
| **N-status** |  |
| N0 (reference) | 103 (46.8%) |
| N1 | 38 (17.3%) |
| HR 1.11; 95% CI 0.537-2.28; p-value = 0.786 (OS) |  |
| N2 | 77 (35.0%) |
| HR 2.21; 95% CI 1.35-3.61; p-value = 0.0016 (OS) | ** |
| N3 | 2 (0.9%) |
| HR 16.3; 95% CI 3.76-70.9; p-value = 0.000194 (OS) | *** |
| **M-status** |  |
| n.d. | 112 |
| M0 | 108 (100.0%) |
| **pEMT-SingScore** |  |
| Median | 0.401 |
| Mean | 0.378 |
| Range | 0.164 - 0.482 |
| HR 44; 95% CI 1.24-1560; p-value = 0.0379 (OS) | * |
| **pEMT-SingScore stratified** |  |
| High (reference) | 88 (40.0%) |
| medium | 44 (20.0%) |
| HR 1.07; 95% CI 0.616-1.86; p-value = 0.806 (OS) |  |
| low | 88 (40.0%) |
| HR 0.574; 95% CI 0.338-0.976; p-value = 0.0405 (OS) | * |
