# Supplementary material for: SLUG‐related partial epithelial‐to‐mesenchymal transition is a transcriptomic prognosticator of head and neck cancer survival
Source: Mol Oncol. 2021 Aug 21;16(2):347–67. doi: 10.1002/1878-0261.13075 (PMC8763659; doi:10.1002/1878-0261.13075)
Supplement: Supplementary file 10 — Table S3. Clinical parameters of the MDACC HNSCC cohort implemented in uni‐ and multivariable analyses. [file MOL2-16-347-s009.docx]

**Supplementary Table 3:** Clinical parameters of the MDACC HNSCC cohort implemented in uni- and multivariable analyses. n.d.: not defined. OS: Overall survival; UICC: Union for International Cancer Control. S: surgery, RT: radiotherapy, cRT: Chemo-radiotherapy, IC: Induction chemotherapy. References for categorical variables are indicated. Significant p-values are indicated: * < 0.05; ** < 0.01; *** < 0.001.

| **Number of patients** | **n = 51** |
| --- | --- |
| **OS (months)** |  |
| Median | 19.934 |
| Mean | 20.786 |
| Range | 0.852 - 60.000 |
| **Age** |  |
| Median | 60.000 |
| Mean | 58.863 |
| Range | 22.000 - 84.000 |
| HR 1; 95% CI 0.974-1.03; p-value = 0.844 (OS) |  |
| **Gender** |  |
| Female (reference) | 12 (23.5%) |
| Male | 39 (76.5%) |
| HR 0.733; 95% CI 0.305-1.76; p-value = 0.488 (OS) |  |
| **Nodal recurrence** |  |
| N0 (reference) | 41 (80.4%) |
| N+ | 10 (19.6%) |
| HR 4.16; 95% CI 1.84-9.44; p-value = 0.000635 (OS) | *** |
| **Local recurrence** |  |
| 0 | 47 (92.2%) |
| 1 | 4 (7.8%) |
| HR 2.04; 95% CI 0.698-5.96; p-value = 0.193 (OS) |  |
| **Extracapsular spread (ECS)** |  |
| N0 (reference) | 35 (68.6%) |
| Yes | 16 (31.4%) |
| HR 2.2; 95% CI 0.959-5.06; p-value = 0.0627 (OS) |  |
| **Primary sites** |  |
| Oral cavity | 49 (96.1%) |
| Oropharynx | 2 (3.9%) |
| **OSCC death** |  |
| N0 | 35 (68.6%) |
| Yes | 16 (31.4%) |
| HR 4.57; 95% CI 2.06-10.1; p-value = 0.000189 (OS) | *** |
| **Perineural invasion** |  |
| 0 (reference) | 23 (45.1%) |
| 1 | 7 (13.7%) |
| HR 0.98; 95% CI 0.205-4.68; p-value = 0.98 (OS) |  |
| 2 | 21 (41.2%) |
| HR 3.36; 95% CI 1.41-7.99; p-value = 0.00627 (OS) | ** |
| **Smoking status** |  |
| Current (reference) | 20 (39.2%) |
| Former | 20 (39.2%) |
| HR 0.638; 95% CI 0.259-1.57; p-value = 0.326 (OS) |  |
| NeverSmoker | 11 (21.6%) |
| HR 0.866; 95% CI 0.324-2.32; p-value = 0.775 (OS) |  |
| **Treatment** |  |
| cRT, S (reference) | 1 (2.0%) |
| IC, S | 1 (2.0%) |
| HR 1.07e+08; 95% CI 0-inf; p-value = 0.998 (OS) |  |
| IC, S, RT | 1 (2.0%) |
| HR 62200000; 95% CI 0-inf; p-value = 0.998 (OS) |  |
| S | 19 (37.3%) |
| HR 29200000; 95% CI 0-inf; p-value = 0.998 (OS) |  |
| S, cRT | 4 (7.8%) |
| HR 28600000; 95% CI 0-inf; p-value = 0.998 (OS) |  |
| S, IC, S, cRT | 1 (2.0%) |
| HR 49400000; 95% CI 0-inf; p-value = 0.998 (OS) |  |
| S, RT | 24 (47.1%) |
| HR 2.5e+07; 95% CI 0-inf; p-value = 0.998 (OS) |  |
| **Stage (UICC)** |  |
| I (reference) | 3 (5.9%) |
| II | 13 (25.5%) |
| HR 686200000; 95% CI 0-inf; p-value = 0.997 (OS) |  |
| III | 11 (21.6%) |
| HR 568200000; 95% CI 0-inf; p-value = 0.997 (OS) |  |
| IV | 24 (47.1%) |
| HR 1.18e+08; 95% CI 0-inf; p-value = 0.997 (OS) |  |
| **pEMT-SingScore** |  |
| Median | 0.192 |
| Mean | 0.166 |
| Range | -0.040 - 0.292 |
| HR 1190000; 95% CI 59.8-2.34e+08; p-value = 0.00256 (OS) | ** |
| **pEMT-SingScore stratified** |  |
| High (reference) | 21 (41.2%) |
| medium | 9 (17.6%) |
| HR 0.352; 95% CI 0.117-1.06; p-value = 0.0643 (OS) |  |
| low | 21 (41.2%) |
| HR 0.267; 95% CI 0.104-0.687 p-value = 0.00614 (OS) | ** |
