# Supplementary material for: SLUG‐related partial epithelial‐to‐mesenchymal transition is a transcriptomic prognosticator of head and neck cancer survival
Source: Mol Oncol. 2021 Aug 21;16(2):347–67. doi: 10.1002/1878-0261.13075 (PMC8763659; doi:10.1002/1878-0261.13075)
Supplement: Supplementary file 11 — Table S4. Clinical parameters of the FHCRC OSCC cohort implemented in uni‐ and multivariable analyses. [file MOL2-16-347-s011.docx]

**Supplementary Table 4:** Clinical parameters of the FHCRC OSCC cohort implemented in uni- and multivariable analyses. OS: Overall survival; UICC: Union for International Cancer Control. References for categorical variables are indicated. Significant p-values are indicated: * < 0.05; ** < 0.01; *** < 0.001.

| **Number of patients** | n = 84 |
| --- | --- |
| **OS (months)** |  |
| Median | 55.380 |
| Mean | 41.928 |
| Range | 0.460 - 60.000 |
| **Gender** |  |
| Female (reference) | 28 (33.3%) |
| Male | 56 (66.7%) |
| HR 1.05; 95% CI 0.534-2.04; p-value = 0.897 (OS) |  |
| **Age** |  |
| 19-39 (reference) | 5 (6.0%) |
| 40-49 | 13 (15.5%) |
| HR 0.864; 95% CI 0.167-4.45; p-value = 0.861 (OS) |  |
| 50-59 | 26 (31.0%) |
| HR 1.47; 95% CI 0.338-6.41; p-value = 0.606 (OS) |  |
| 60-88 | 40 (47.6%) |
| HR 0.862; 95% CI 0.197-3.77; p-value = 0.844 (OS) |  |
| **Tissue** |  |
| HPV-negative oral squamous cell carcinoma (OSCC) | 84 (100.0%) |
| **Stage (UICC)** |  |
| I/II (reference) | 37 (44.0%) |
| III/IV | 47 (56.0%) |
| HR 4.26; 95% CI 1.95-9.31; p-value = 0.000286 (OS) | *** |
| **Treatment** |  |
| multi-modality (reference) | 45 (53.6%) |
| uni-modality | 39 (46.4%) |
| HR 0.58; 95% CI 0.299-1.12; p-value = 0.105 (OS) |  |
| **pEMT-SingScore** |  |
| Median | 0.327 |
| Mean | 0.312 |
| Range | 0.095 - 0.399 |
| HR 654 95% CI 2.51-170000; p-value = 0.0224 (OS) | * |
| **pEMT-SingScore stratified** |  |
| High (reference) | 34 (40.5%) |
| medium | 16 (19.0%) |
| HR 0.641; 95% CI 0.274-1.5; p-value = 0.306 (OS) |  |
| low | 34 (40.5%) |
| HR 0.29; 95% CI 0.133-0.631; p-value = 0.800183 (OS) | ** |
