# Supplementary material for: SLUG‐related partial epithelial‐to‐mesenchymal transition is a transcriptomic prognosticator of head and neck cancer survival
Source: Mol Oncol. 2021 Aug 21;16(2):347–67. doi: 10.1002/1878-0261.13075 (PMC8763659; doi:10.1002/1878-0261.13075)
Supplement: Supplementary file 12 — Table S5. Gene lists of up‐ and down‐regulated genes in the TCGA, MDACC and FHCRC cohorts identified using DESeq, edgeR and limma/limma‐voom algorithms and pEMT‐Singscorelow patients (lower 40%) as baseline to compare with pEMT‐Singscorehigh patients (upper 40%). [file MOL2-16-347-s002.docx]

**Supplementary Table 5:** Gene lists of up- and down-regulated genes in the TCGA, MDACC and FHCRC cohorts identified using DESeq, edgeR and limma/limma-voom algorithms and pEMT-Singscore^low^ patients (lower 40%) as baseline to compare with pEMT-Singscore^high^ patients (upper 40%). Regulated genes are the intersection of all cohorts and algorithms. DEGs were compared with all six gene signatures described in Puram *et al.* (Cell, 2017) and the corresponding genes are indicated with a “x”.

| **DEGs Up** | **Cell cycle** | **pEMT** | **EpiDif1** | **EpiDif2** | **Stress** | **Hypoxia** |
| --- | --- | --- | --- | --- | --- | --- |
| ABL2 | - | - | - | - | - | - |
| ACOT9 | - | - | - | - | - | - |
| ACTN1 | - | x | - | - | - | - |
| ADAM12 | - | - | - | - | - | - |
| ADAMTS12 | - | - | - | - | - | - |
| AMIGO2 | - | - | - | - | - | - |
| ANXA5 | - | x | - | - | - | - |
| APBB2 | - | - | - | - | - | - |
| APP | - | x | - | - | - | - |
| ARL4C | - | - | - | - | - | - |
| BEND6 | - | - | - | - | - | - |
| BGN | - | - | - | - | - | - |
| CD109 | - | - | - | - | - | - |
| CHST11 | - | - | - | - | - | - |
| CHST15 | - | - | - | - | - | - |
| CLSTN1 | - | - | - | - | - | - |
| COL12A1 | - | - | - | - | - | - |
| COL17A1 | - | x | - | - | - | - |
| COL1A1 | - | x | - | - | - | - |
| COL4A6 | - | - | - | - | - | - |
| COL5A1 | - | - | - | - | - | - |
| COL5A2 | - | x | - | - | - | - |
| CSPG4 | - | - | - | - | - | - |
| CTSV | - | - | - | - | - | - |
| DCBLD2 | - | - | - | - | - | - |
| DKK3 | - | x | - | - | - | - |
| EMC1 | - | - | - | - | - | - |
| EVA1A | - | - | - | - | - | - |
| EXT2 | - | x | - | - | - | - |
| F2R | - | - | - | - | - | - |
| F3 | - | x | - | - | - | - |
| FKBP14 | - | - | - | - | - | - |
| FKBP9 | - | x | - | - | - | - |
| FN1 | - | - | - | - | - | - |
| GALNT10 | - | - | - | - | - | - |
| GALNT2 | - | x | - | - | - | - |
| GJA1 | - | x | - | - | - | - |
| GPR39 | - | - | - | - | - | - |
| GPX8 | - | - | - | - | - | - |
| HTRA1 | - | x | - | - | - | - |
| IKBIP | - | - | - | - | - | - |
| IL11 | - | - | - | - | - | - |
| INHBA | - | x | - | - | - | - |
| ITGA6 | - | x | - | - | - | - |
| ITGAV | - | - | - | - | - | - |
| IVNS1ABP | - | - | - | - | - | - |
| KDELR3 | - | - | - | - | - | - |
| LAMA3 | - | x | - | - | - | - |
| LAMB1 | - | - | - | - | - | - |
| LAMB3 | - | x | - | - | x | - |
| LAMC2 | - | x | - | - | - | - |
| LOXL2 | - | - | - | - | - | - |
| LPCAT1 | - | - | - | - | - | - |
| MFAP2 | - | x | - | - | - | - |
| MICAL2 | - | - | - | - | - | - |
| MMP1 | - | x | - | - | - | - |
| MMP10 | - | x | - | - | - | - |
| MMP9 | - | - | - | - | - | - |
| MYH9 | - | x | - | - | - | - |
| MYO1B | - | - | - | - | - | - |
| NEK6 | - | - | - | - | - | - |
| NRP2 | - | - | - | - | - | - |
| PDGFC | - | - | - | - | - | - |
| PDPN | - | x | - | - | - | - |
| PLAU | - | x | - | - | - | x |
| PLEK2 | - | - | - | - | - | - |
| PLOD2 | - | x | - | - | - | x |
| PMEPA1 | - | - | - | - | - | - |
| PRSS23 | - | x | - | - | - | - |
| PTGFRN | - | - | - | - | - | - |
| PTHLH | - | x | - | - | - | x |
| PTK7 | - | x | - | - | - | - |
| RTTN | - | - | - | - | - | - |
| SERPINE1 | - | x | - | - | - | x |
| SERPINE2 | - | x | - | - | - | - |
| SERPINH1 | - | x | - | - | - | - |
| SFXN3 | - | - | - | - | - | - |
| SKIL | - | - | - | - | - | - |
| SLC39A14 | - | x | - | - | - | - |
| SMIM3 | - | - | - | - | - | - |
| SNAI2 | - | - | - | - | - | - |
| SORL1 | - | - | - | - | - | - |
| SUGCT | - | - | - | - | - | - |
| TES | - | - | - | - | - | - |
| TGFBI | - | x | - | - | - | - |
| TNFAIP6 | - | - | - | - | - | - |
| TNFRSF12A | - | x | - | - | - | - |
| TRIO | - | - | - | - | - | - |
| TSHZ3 | - | - | - | - | - | - |

| **DEGs Down** | **Cell cycle** | **pEMT** | **EpiDif1** | **EpiDif2** | **Stress** | **Hypoxia** |
| --- | --- | --- | --- | --- | --- | --- |
| PPFIBP2 | - | - | - | - | - | - |
| PPM1L | - | - | - | - | - | - |
| TRIM7 | - | - | - | - | - | - |
