# Supplementary material for: SLUG‐related partial epithelial‐to‐mesenchymal transition is a transcriptomic prognosticator of head and neck cancer survival
Source: Mol Oncol. 2021 Aug 21;16(2):347–67. doi: 10.1002/1878-0261.13075 (PMC8763659; doi:10.1002/1878-0261.13075)
Supplement: Supplementary file 13 — Table S6. Clinical parameters of the LMU HNSCC cohort implemented in uni‐ and multivariable analyses. [file MOL2-16-347-s003.docx]

**Supplementary Table 6:** Clinical parameters of the LMU HNSCC cohort implemented in uni- and multivariable analyses. n.d.: not defined. OS: overall survival; DFS: Disease-free survival. HR: Hazard ratio; 95% CI: 95% confidence interval. References for variables are indicated. Significant p-values are indicated: *** < 0.001.

| **Number of patients** | n = 76 |
| --- | --- |
| **OS (months)** |  |
| Median | 18.97 |
| Mean | 22.56 |
| Range | 1.12 - 60.00 |
| **DFS (months)** |  |
| Median | 14.69 |
| Mean | 19.37 |
| Range | 1.12 - 60.00 |
| **Treatment** |  |
| Surgery (curative) | 76 (100%) |
| Adjuvant therapy  (radiotherapy, chemoradiotherapy, immunoradiotherapy) | 52 (68.4 %) |
| HR 0.333; 95% CI 0.126-0879; p-value = 0.0265 (DFS) | * |
| **Primary site** |  |
| Hypopharynx & Larynx | 16 (21.05%) |
| HR 1.09; 95% CI 0.426-2.78; p-value = 0.86 (DFS) |  |
| Oral cavity (reference) | 25 (32.89%) |
| Oropharynx | 35 (46.05%) |
| HR 0.697; 95% CI 0.307-1.58; p-value = 0.389 (DFS) |  |
| **T-stage** |  |
| T1 | 5 (6.6%) |
| T2 | 27 (35.5%) |
| T3 | 33 (43.5%) |
| T4 | 10 (13.1%) |
| pTx | 1 (1.3%) |
| **N-stage** |  |
| N0 (reference) | 29 (38.15%) |
| N1 | 5 (6.58%) |
| HR 0.506; 95% CI 0.0666-3.85; p-value = 0.511 (DFS) |  |
| N2 | 13 (17.10%) |
| HR 0.84; 95% CI 0.241-2.93; p-value = 0.785 (DFS) |  |
| N3 | 19 (25%) |
| HR 0.933; 95% CI 0.39-2.23; p-value = 0.876 (DFS) |  |
| Nx | 10 (13.15%) |
| **Extranodal extension** |  |
| N0 (reference) | 29 (38.15%) |
| ENE- | 16 (21.05%) |
| HR 0.449; 95% CI 0.16-1.34; p-value = 0.152 (DFS) |  |
| ENE+ | 19 (25.0%) |
| HR 0.862; 95% CI 0.368-2.02; p-value = 0.732 (DFS) |  |
| n.d. | 12 (15.78%) |
| **Lymphovascular invasion** |  |
| L0 | 43 (56.6%) |
| HR 1100000; 95% CI 0-inf; p-value = 0.997 (DFS) |  |
| L1 | 28 (36,8%) |
| HR 1490000; 95% CI 0-inf; p-value = 0.997 (DFS) |  |
| n.d. (reference) | 5 (6,6%) |
| **Angioinvasion** |  |
| V0 | 66 (86.8%) |
| HR 1220000; 95% CI 0-inf; p-value = 0.997 (DFS) |  |
| V1 | 5 (6.6%) |
| HR 1620000; 95% CI 0-inf; p-value = 0.997 (DFS) |  |
| n.d. (reference) | 5 (6.6%) |
| **Perineural invasion** |  |
| Pn0 | 47 (61.8%) |
| HR 0.633; 95% CI 0.179-2.24; p-value = 0.479 (DFS) |  |
| Pn1 | 22 (28.9%) |
| HR 3.01; 95% CI 0.865-10.5; p-value = 0.0833 (DFS) |  |
| n.d. (reference) | 7 (9.2%) |
| **Grading** |  |
| G1 | 1 (1.31%) |
| HR 1.6e+08; 95% CI 0-inf; p-value = 1 (DFS) |  |
| G2 | 28 (36.84%) |
| HR 41200000; 95% CI 0-inf; p-value = 1 (DFS) |  |
| G3 | 44 (57.89%) |
| HR 41800000; 95% CI 0-inf; p-value = 1 (DFS) |  |
| n.d. | 3 (3.95%) |
| **Recurrence** |  |
| yes | 26 (34.2%) |
| HR 11.6; 95% CI 4.99-26.8; p-value = 1.17e-08 (DFS) | *** |
| no (reference) | 50 (65.8%) |
| **SLUG IHC score** |  |
| HR 1.01; CI 1.000104 - 1.010123; p-value = 0.0454 | * |
