# Supplementary material for: SLUG‐related partial epithelial‐to‐mesenchymal transition is a transcriptomic prognosticator of head and neck cancer survival
Source: Mol Oncol. 2021 Aug 21;16(2):347–67. doi: 10.1002/1878-0261.13075 (PMC8763659; doi:10.1002/1878-0261.13075)
Supplement: Supplementary file 14 — Table S7. Clinical parameters of the matched triplets of normal mucosa, primary tumor and lymph node metastases analyzed for Slug expression. [file MOL2-16-347-s008.docx]

**Supplementary Table 7:** Clinical parameters of the matched triplets of normal mucosa, primary tumor and lymph node metastases analyzed for Slug expression.

| **Number of patients** | n = 15 |
| --- | --- |
| **T-stage** |  |
| T1 | 1 (6.3%) |
| T2 | 6 (40.0%) |
| T3 | 5 (33.3%) |
| T4 | 3 (20.0%) |
| **N-stage** |  |
| N1 | 3 (20.0%) |
| N2a | 1 (6.3%) |
| N2b | 4 (26.7%) |
| N3a | 1 (6.3%) |
| N3b | 6 (40.0%) |
| **HPV status** |  |
| HPV- | 15 (100.0%) |
| **Extranodal extension** |  |
| ENE- | 9 (60.0%) |
| ENE+ | 6 (40.0%) |
| **Lymphovascular invasion** |  |
| L0 | 9 (60.0%) |
| L1 | 6 (40.0%) |
| **Angioinvasion** |  |
| V0 | 15 (100.0%) |
| V1 | 0 |
| **Perineural invasion** |  |
| Pn0 | 12 (80.0%) |
| Pn1 | 3 (20.0%) |
| **Grading** |  |
| G2 | 11 (73.3%) |
| G3 | 4 (26.7%) |
| **Localization** |  |
| Oral cavity | 3 (20.0%) |
| Oropharynx | 6 (40.0%) |
| Hypopharynx | 4 (26.7%) |
| Larynx | 2 (13.3%) |
